# Supplementary figures and images for: Isolating single cycles of neural oscillations in population spiking
Source: PLoS Comput Biol. 2025 Jun 4;21(6):e1013084. doi: 10.1371/journal.pcbi.1013084 (PMC12136316; doi:10.1371/journal.pcbi.1013084)

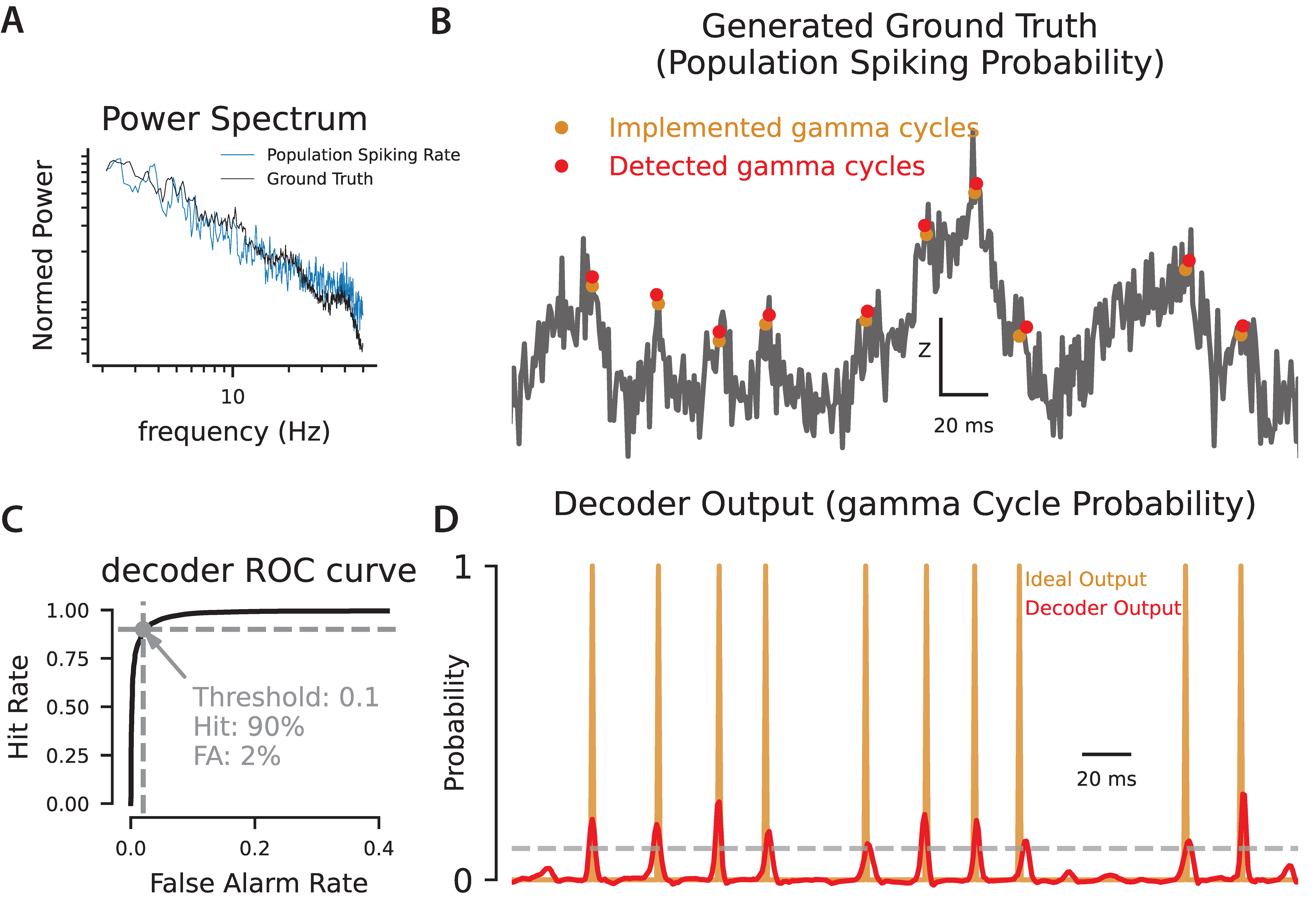

Supplement: S1 Fig — A. The blue trace represents the average power spectrum of the population spiking rate from 29 recording sites (4 S1 recording sites, 5 V1 recording sites, and 22 recording sites across the brain in Mouse 1 from [26]). The black trace is the power spectrum of the generated ground-truth. The ground truth was generated using six cycle types (high gamma, gamma, beta, alpha, theta, and delta see Fig 1A). Each cycle type occurred with a 60% chance. The width of each cycle at each instance was randomly jittered by adding a value derived from a normal distribution with μ=0 and σ equal to 30% of the cycle width (e.g. 6.6 ms for the gamma cycle). After normalizing the signal to range between 0 and 1, the noise was introduced by adding a random value, derived from a normal distribution with μ=0 and σ=0.05, at each time point. B. An example period of the ground truth spiking probability. The decoder was run to detect the gamma cycles on this signal. The orange dots show the center of each implemented gamma cycle in the signal, and the red dots are the detected gamma cycles after thresholding and peak detection on the output of the decoder (see the next panels). C. The decoder’s ROC curve with the threshold used for the cycle detection. D. The decoder output (red trace) in response to the signal in B is compared to the ideal output (orange trace), which is zero everywhere except at the center of the gamma cycles. The dashed line is the threshold level (panel C) chosen to decide the cycle time based on the decoder output. (TIFF) [file pcbi.1013084.s001.tif]

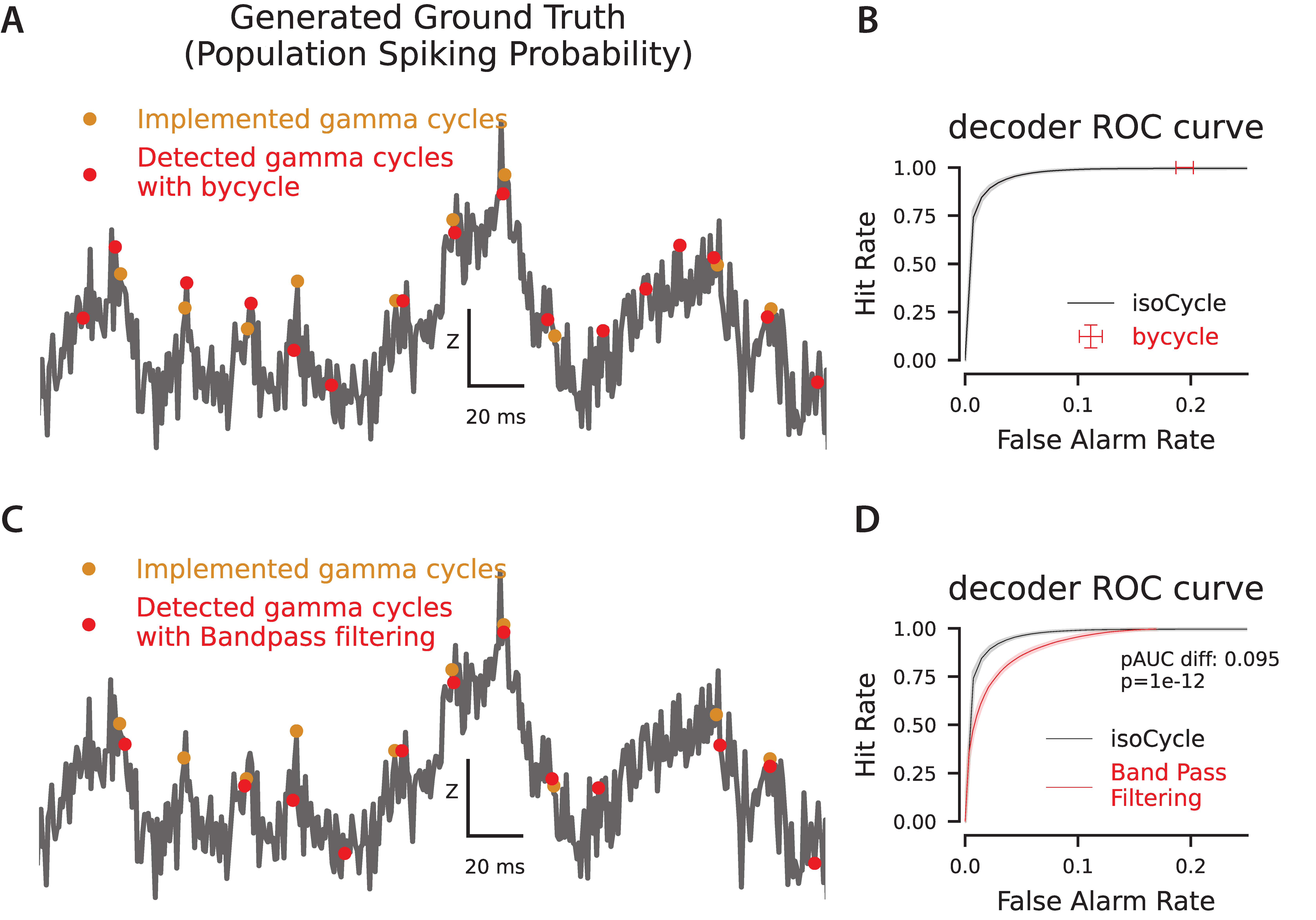

Supplement: S2 Fig — A. An example period of the ground truth spiking probability (same as S1B Fig). The bycyle method [16] was run to detect the gamma cycles on this signal. The orange dots show the center of each implemented gamma cycle in the signal, and the red dots are the detected gamma cycles. The implemented gamma cycles were on average 20 ms in duration. The original signal was low pass filtered, using neurodsp.filt.filter_signal with f_lowpass=150, n_seconds=0.1 and then was analyzed with bycycle.cyclepoints.find_extrema using the following parameters: fs=1280, f_range=(40,60), filter_kwargs=’n_seconds’:0.1. B. The average ROC curve was calculated by using 10 synthetic signals. bycycle doesn’t provide different outputs based on varying thresholds, so the range of the ROC curve for the bycycle is limited. C. Similar to A, red dots indicate the signal’s peaks when bandpass filtered between 40 and 60 Hz. D. Similar to B for comparison of the ROC curve between our method and bandpass filtering while using varying thresholds to identify the signals’ peaks. The partial Area Under the Curve (pAUC) of the ROC for both methods was calculated by normalizing to each method’s maximum False Alarm rate, as their ROC curves are bounded. isoCycle: 0.975 ± 0.002 and band pass filtering: 0.879 ± 0.005. Shaded areas and error bars show standard deviations. (TIFF) [file pcbi.1013084.s002.tif]

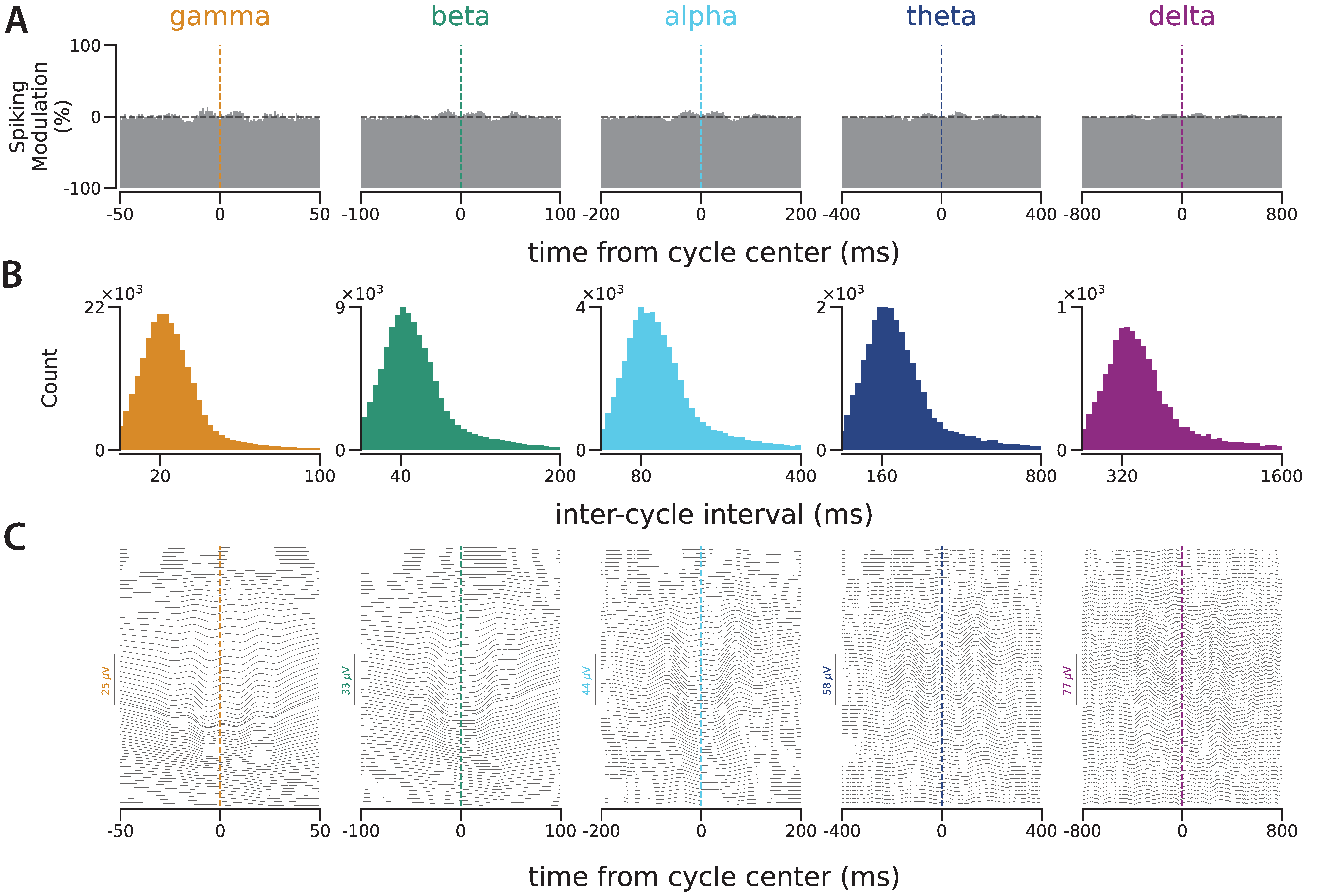

Supplement: S3 Fig — Same analyses as in panels D–F of Fig 1, when a random jitter of up to half a cycle width is added to or subtracted from each detected cycle time. (TIFF) [file pcbi.1013084.s003.tif]

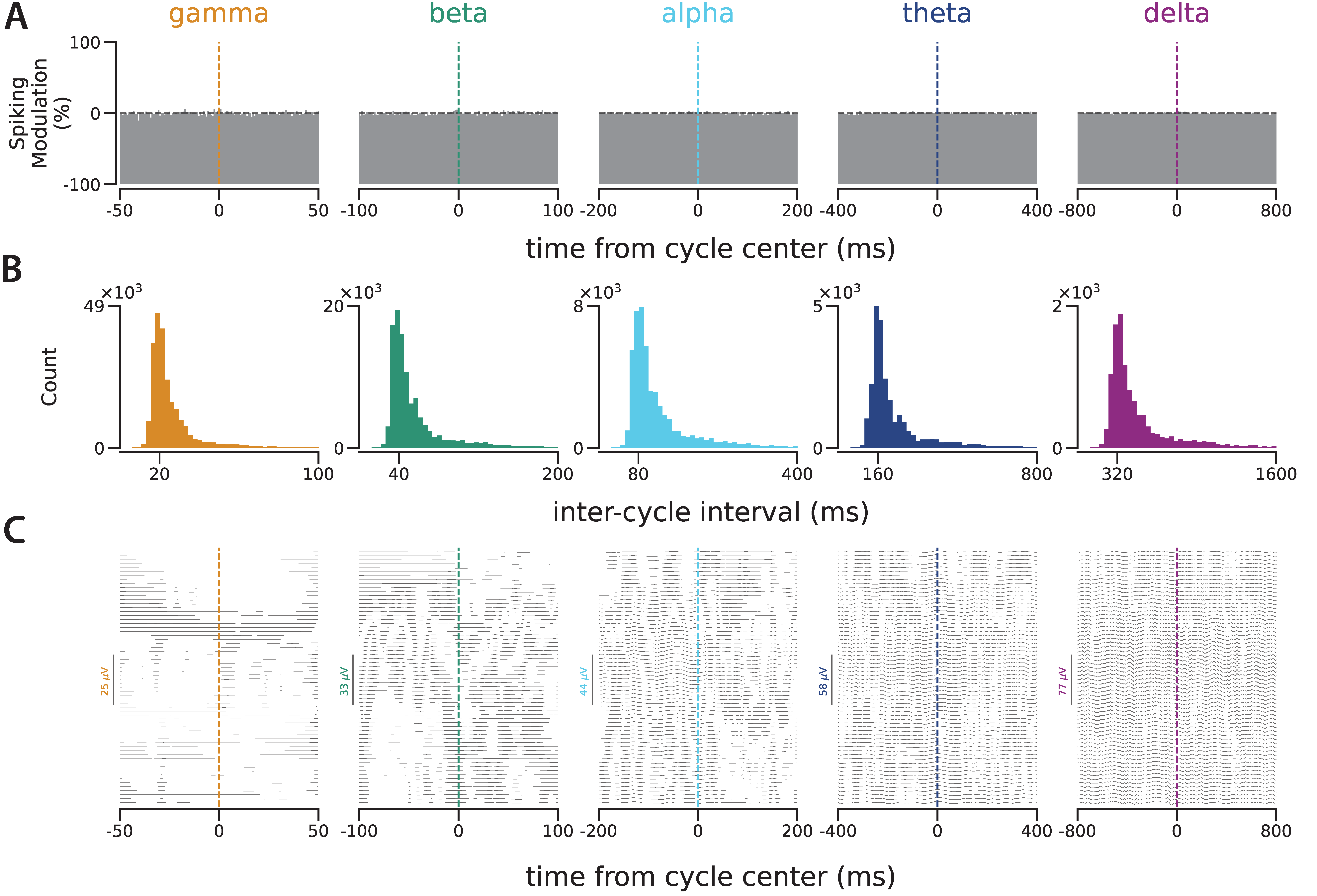

Supplement: S4 Fig — Same analyses as in panels D–F of Fig 1, when the detected cycle times were shuffled while the distribution of the inter-cycle intervals has been kept the same as for the original cycle times. (TIFF) [file pcbi.1013084.s004.tif]

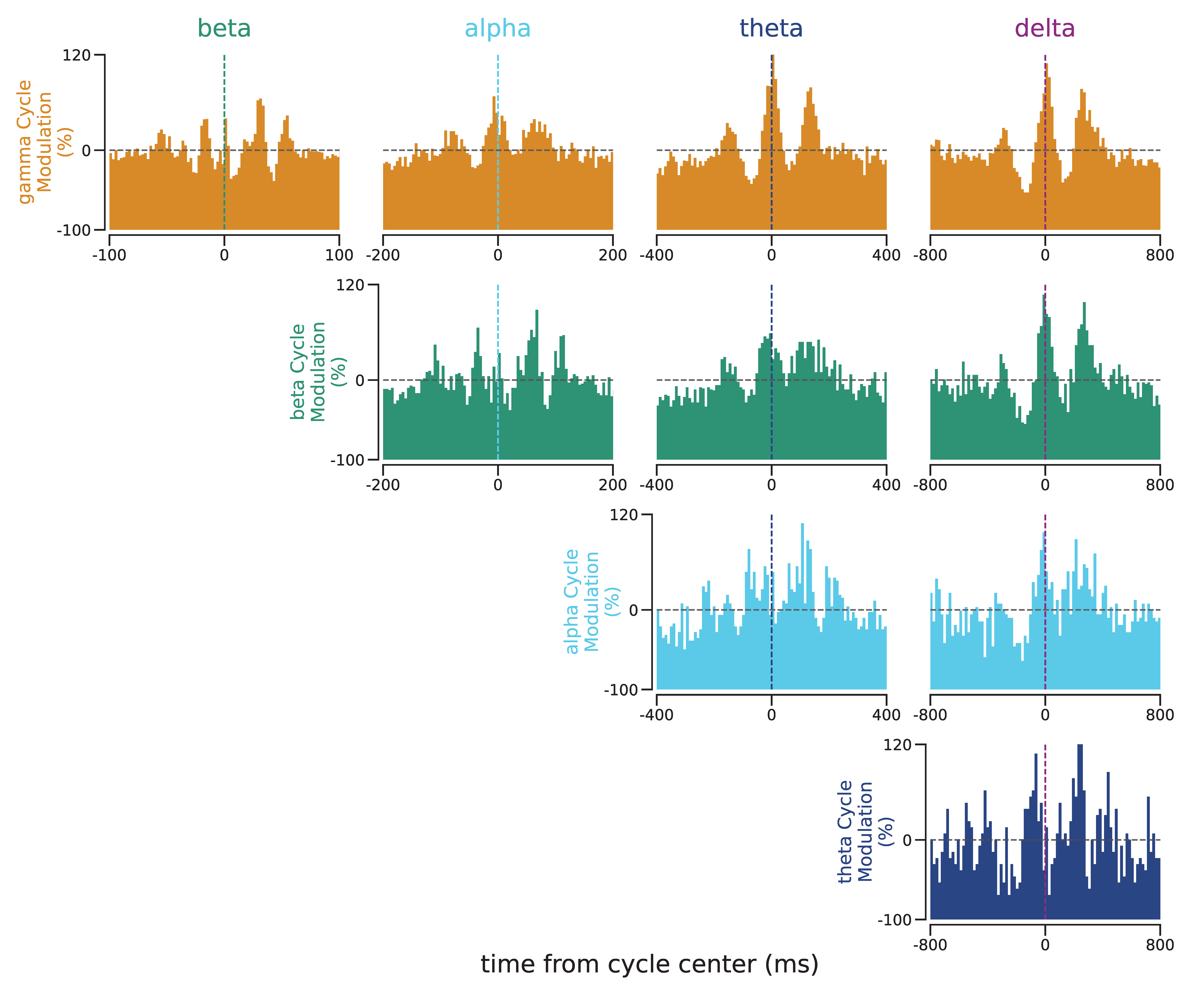

Supplement: S5 Fig — Similar to Fig 1G, between all pairs of cycles (p = 0.5 used as the threshold level for cycle detection in this figure to reduce the chance of false alarms). (TIFF) [file pcbi.1013084.s005.tif]

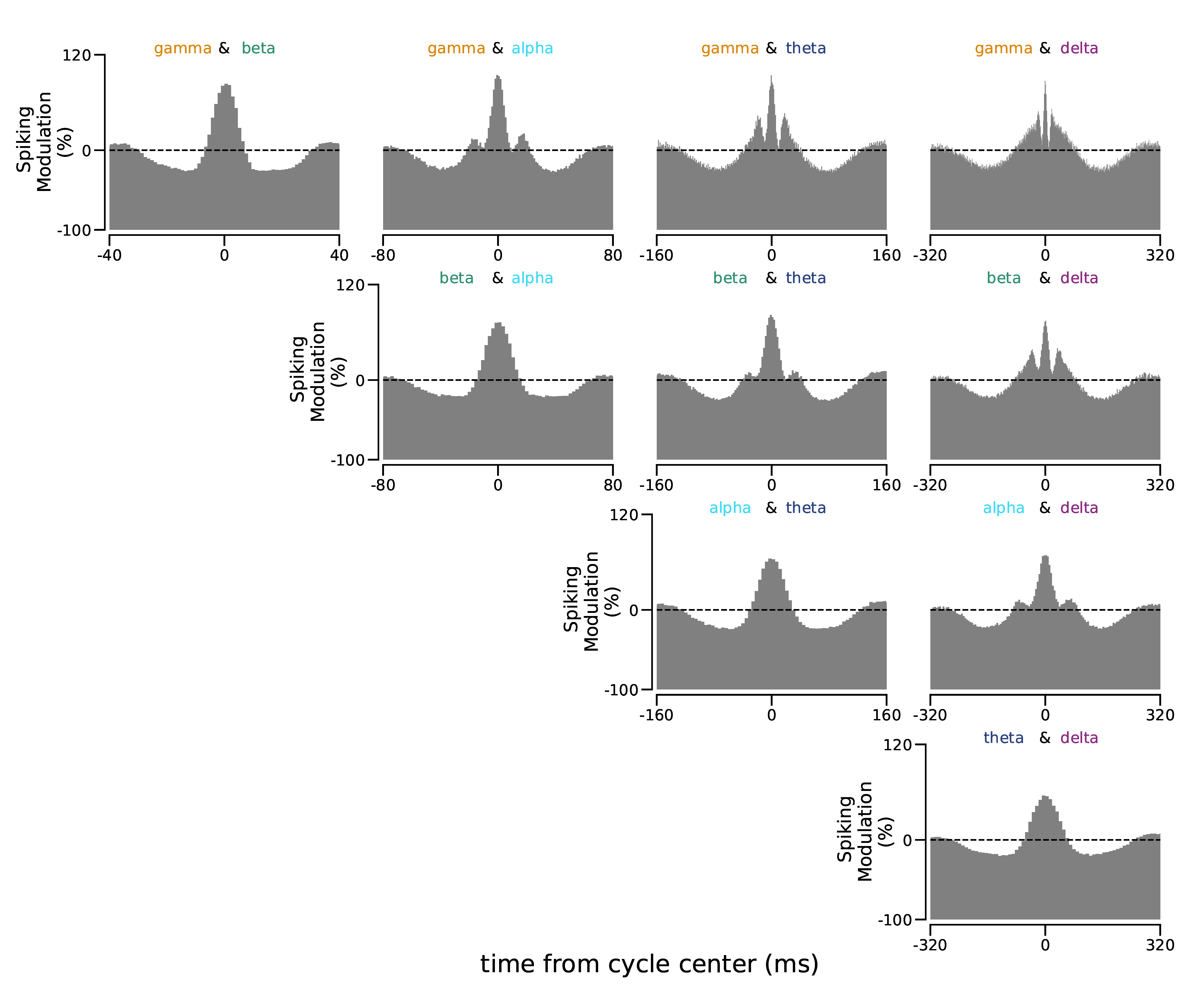

Supplement: S6 Fig — Similar to Fig 1H, with times of co-occurrence for all pairs of cycles (p = 0.1 used as the threshold level for cycle detection in this figure). (TIFF) [file pcbi.1013084.s006.tif]

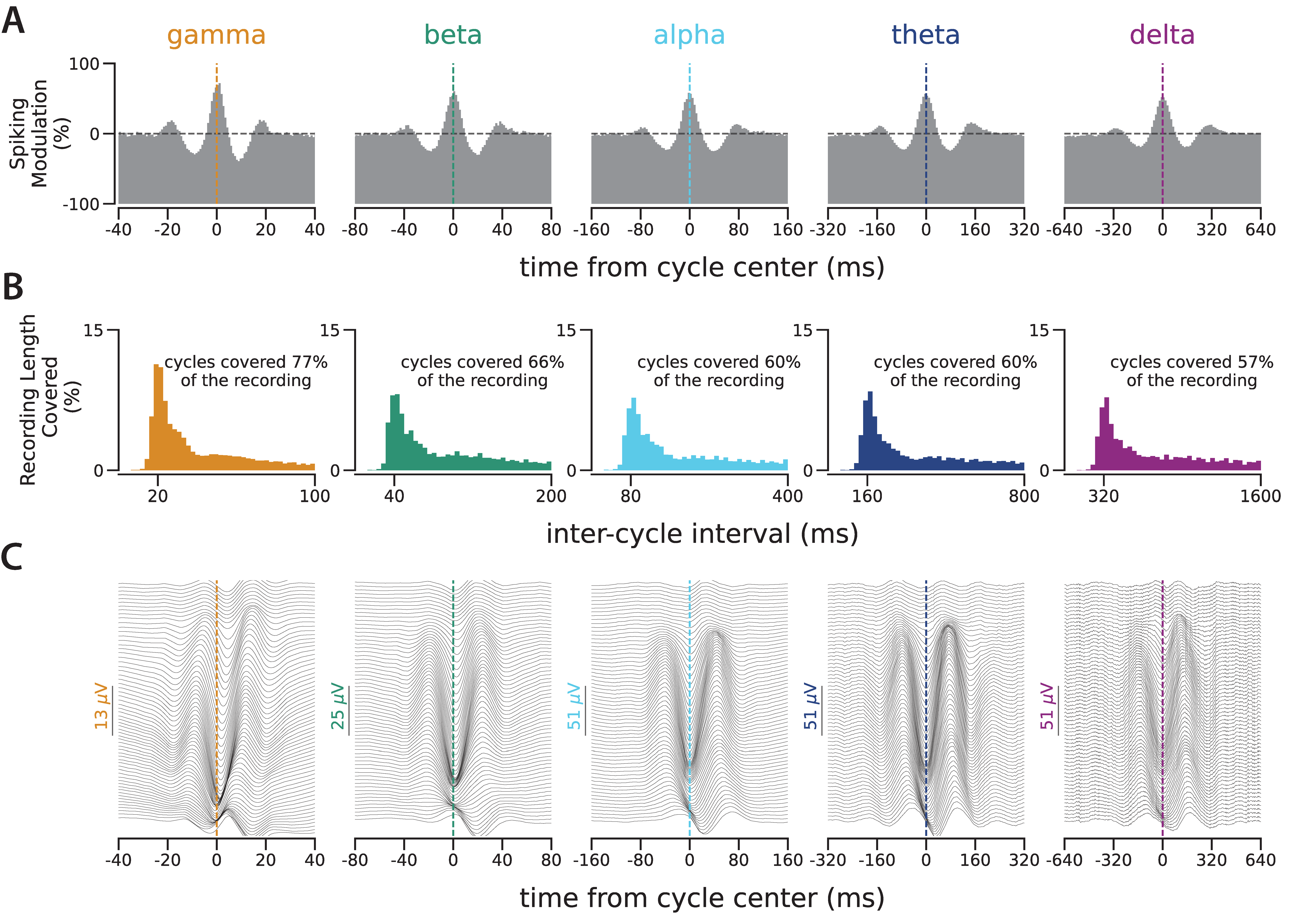

Supplement: S7 Fig — Similar to Fig 1D–1E, for cycles detected from 49 neurons recorded simultaneously in mouse S1 for 8370 seconds. (TIFF) [file pcbi.1013084.s007.tif]

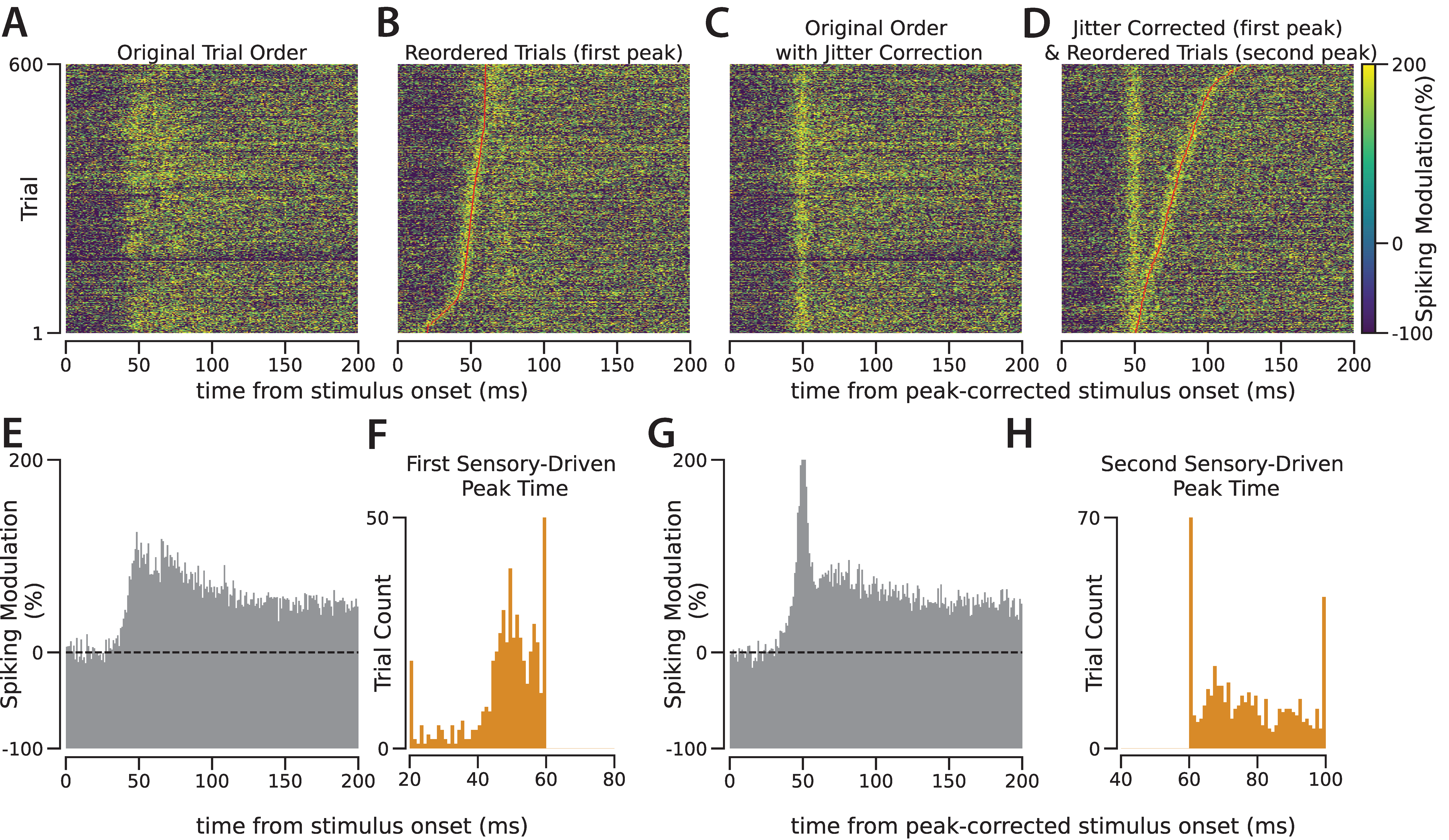

Supplement: S8 Fig — Spiking response to visual stimulation where each 1-ms time bin depicts the combined spiking modulation from 131 simultaneously recorded V1 neurons. Each row represents a trial of visual stimulation, with spiking values relative to the 200-ms pre-stimulus window average. B. As in A, the trials are reordered according to the timing of the peak of the population firing rate in the 20 to 60 ms window after the stimulus onset in each trial. Red dots on each row indicate the time of the peaks in each trial. C. As in A, with the stimulus onset time adjusted based on varying peak timings across trials. D. As in C, the trials are reordered according to the timing of the peak of the population firing rate in the 60 and 100 ms window after the stimulus onset. Red dots on each row indicate the time of the peaks. E. Spiking average across trials, as shown in A. The y-axis shows the modulation of the population spiking relative to the baseline (represented by the dashed line). F. Distribution of the timing of the first sensory-driven peak across trials, relative to stimulus onset. G. As in E, but after adjusting stimulus onset based on varying peak timings across trials. H. Distribution of the timing of the second sensory-driven peak across trials, relative to stimulus onset. (TIFF) [file pcbi.1013084.s008.tif]

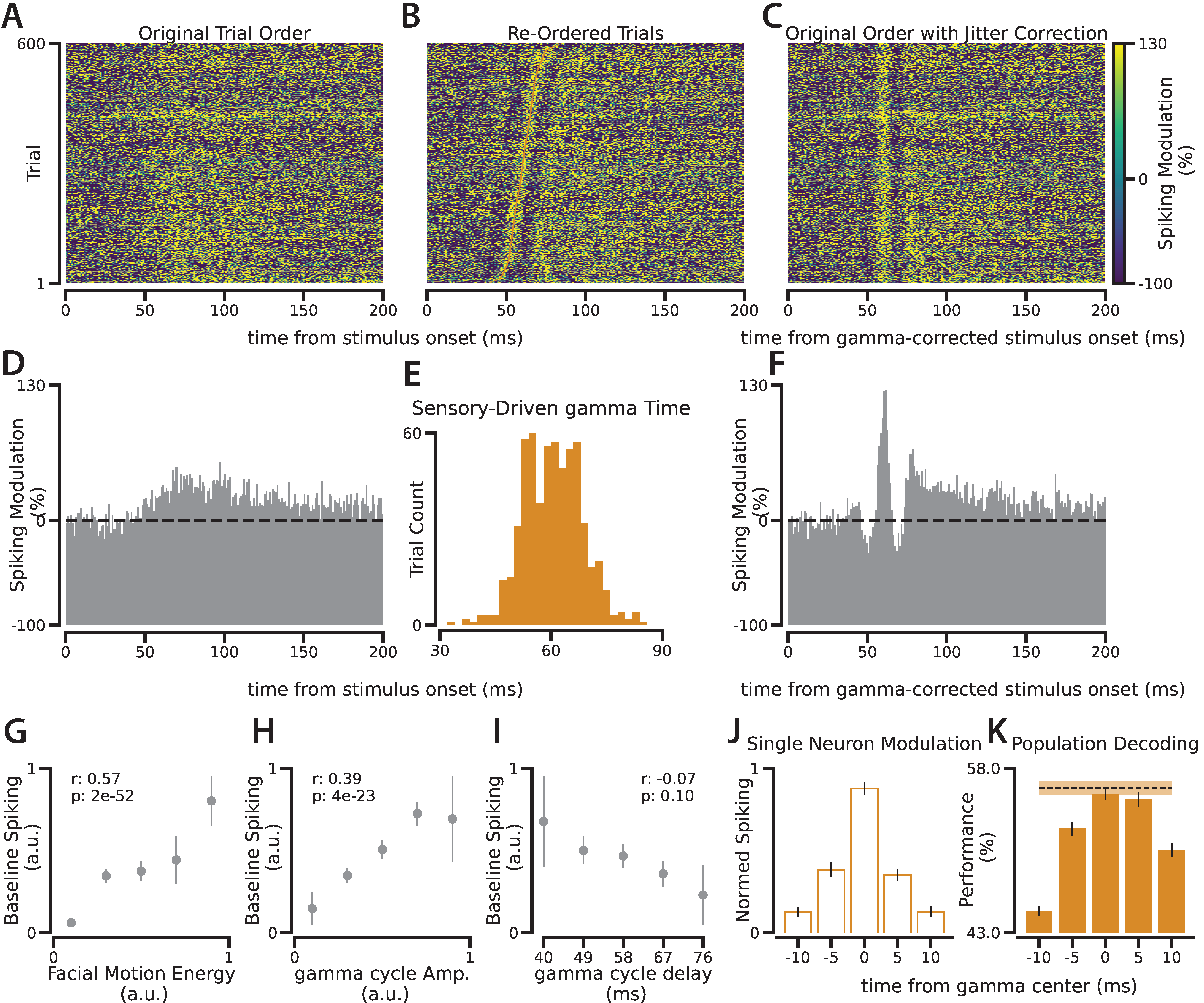

Supplement: S9 Fig — Similar analyses as in Fig 2 for a recording in another animal. In this recording session, 58 neurons were simultaneously recorded while drifting gratings were shown to the animal. (TIFF) [file pcbi.1013084.s009.tif]

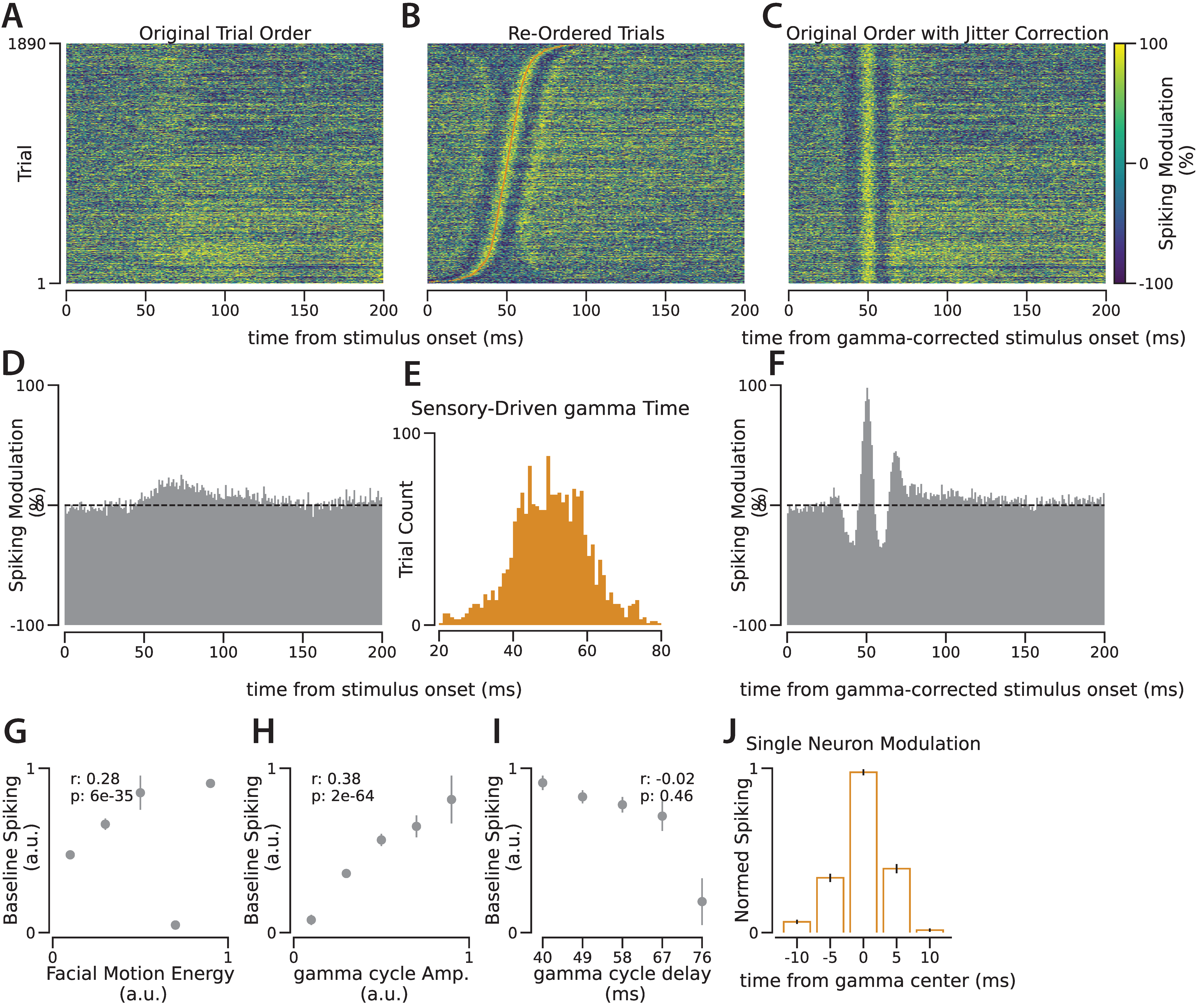

Supplement: S10 Fig — Similar analyses as in Fig 2 for a recording in another animal. In this recording session, 56 neurons have been simultaneously recorded, while 70 distinct natural stimuli during 1890 trials have been shown to the animal. (TIFF)vspace*6pt [file pcbi.1013084.s010.tif]

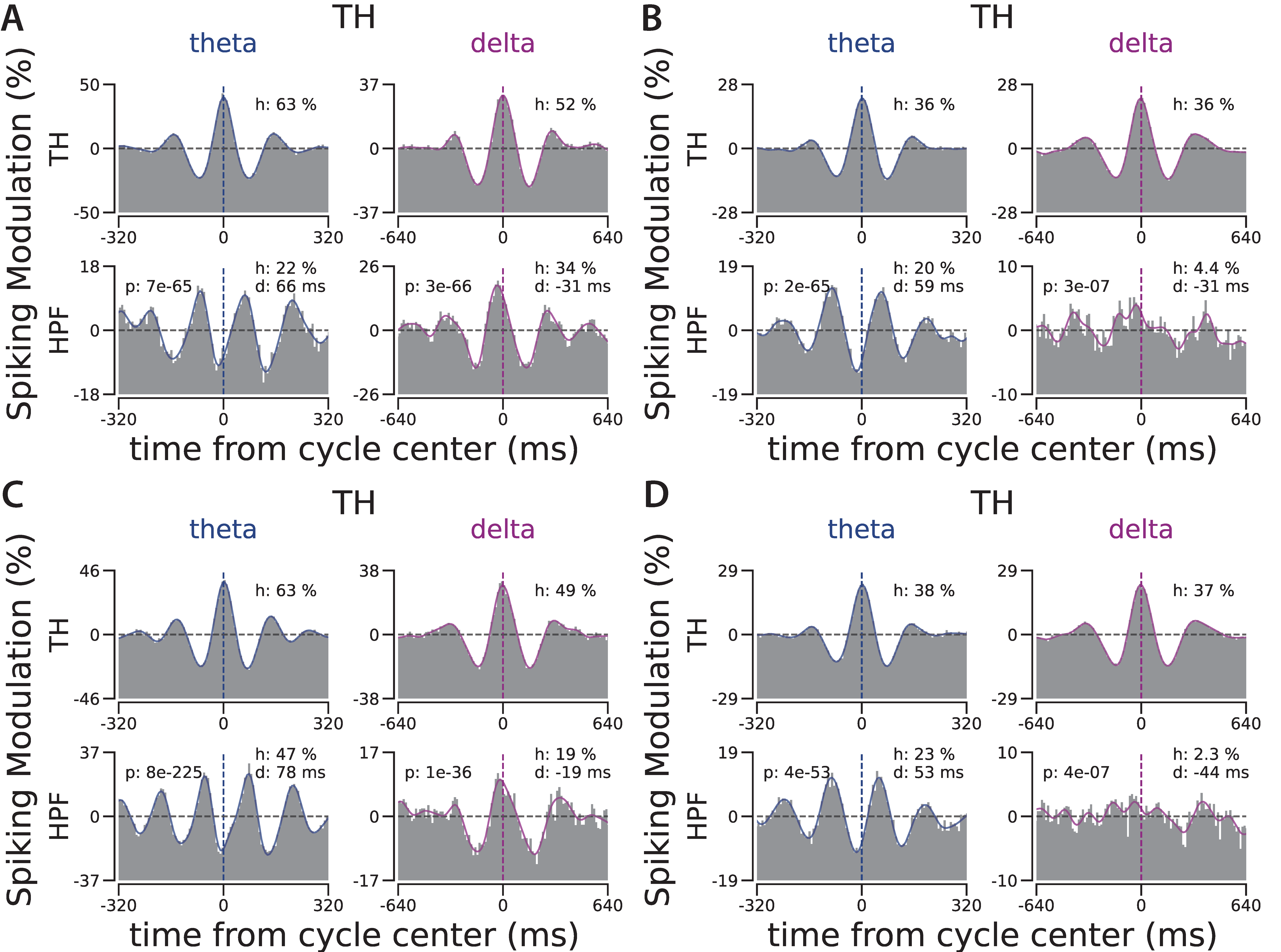

Supplement: S11 Fig — Similar to Fig 3A–3B when the cycles detected in each session’s first and second half have been used separately to compute the spiking distribution. A–B. First half. C–D. Second half. (TIFF) [file pcbi.1013084.s011.tif]

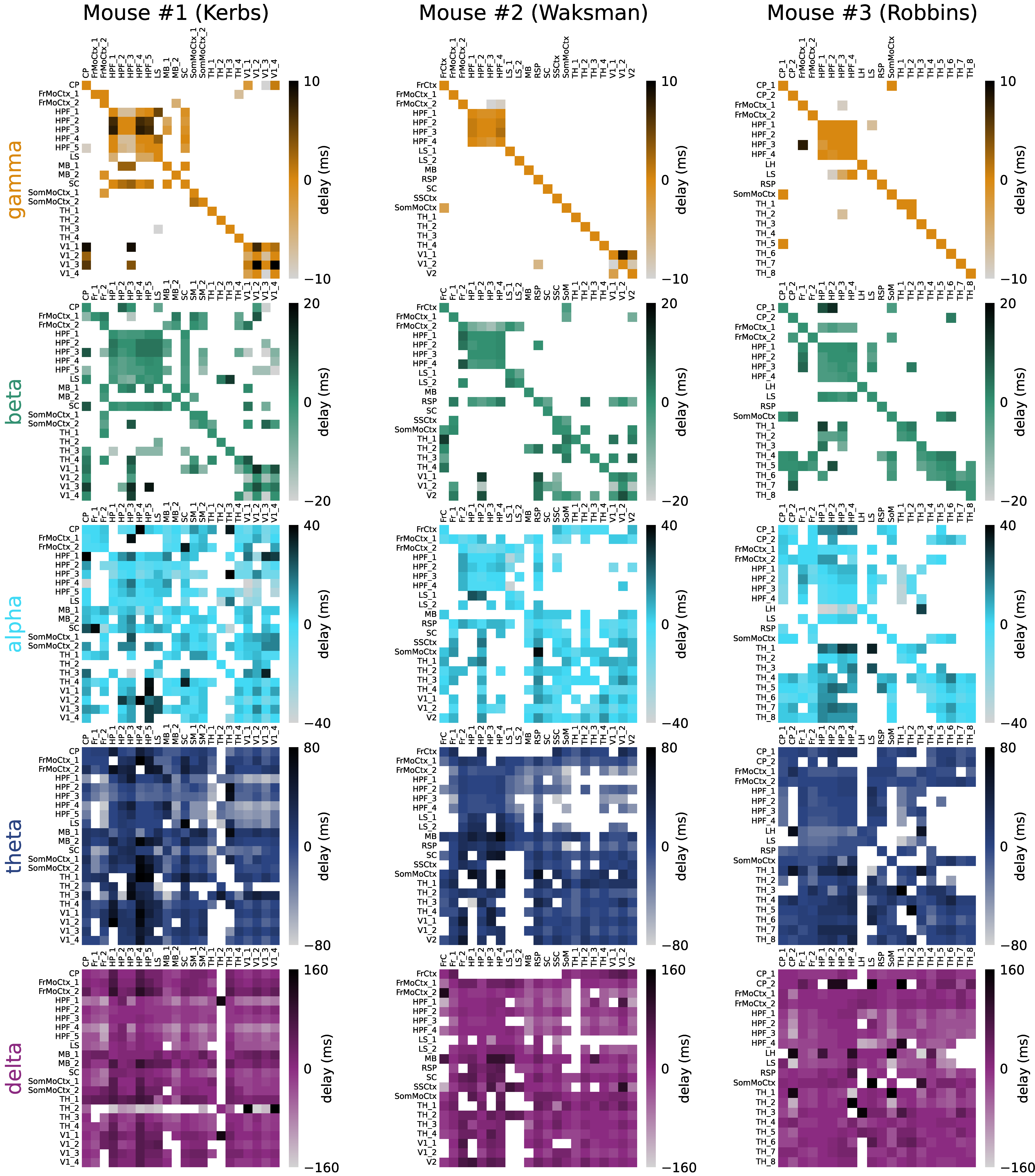

Supplement: S15 Fig — Similar to Fig 3C for gamma, beta, alpha, theta, and delta cycles across all regions in 3 animals. If the pattern of correlated activity between two regions is not significant (see Methods), then the corresponding cell in the delay matrix is left blank. Patterns of correlated spiking across regions, from which these delays were derived, can be found at [61]. (TIFF) [file pcbi.1013084.s015.tif]

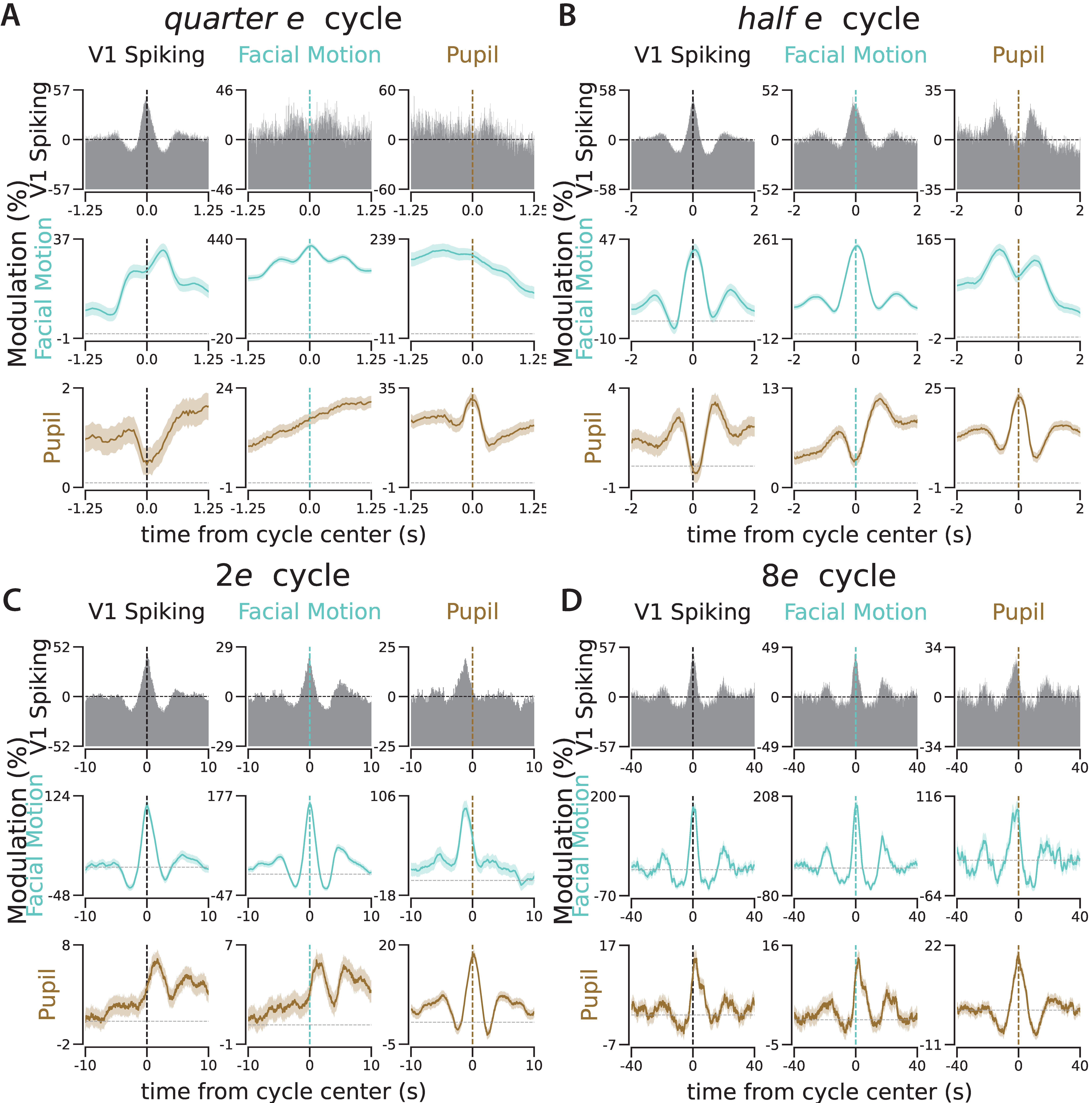

Supplement: S16 Fig — Similar to Fig 4B & 4E for four other slow cycles. (TIFF) [file pcbi.1013084.s016.tif]

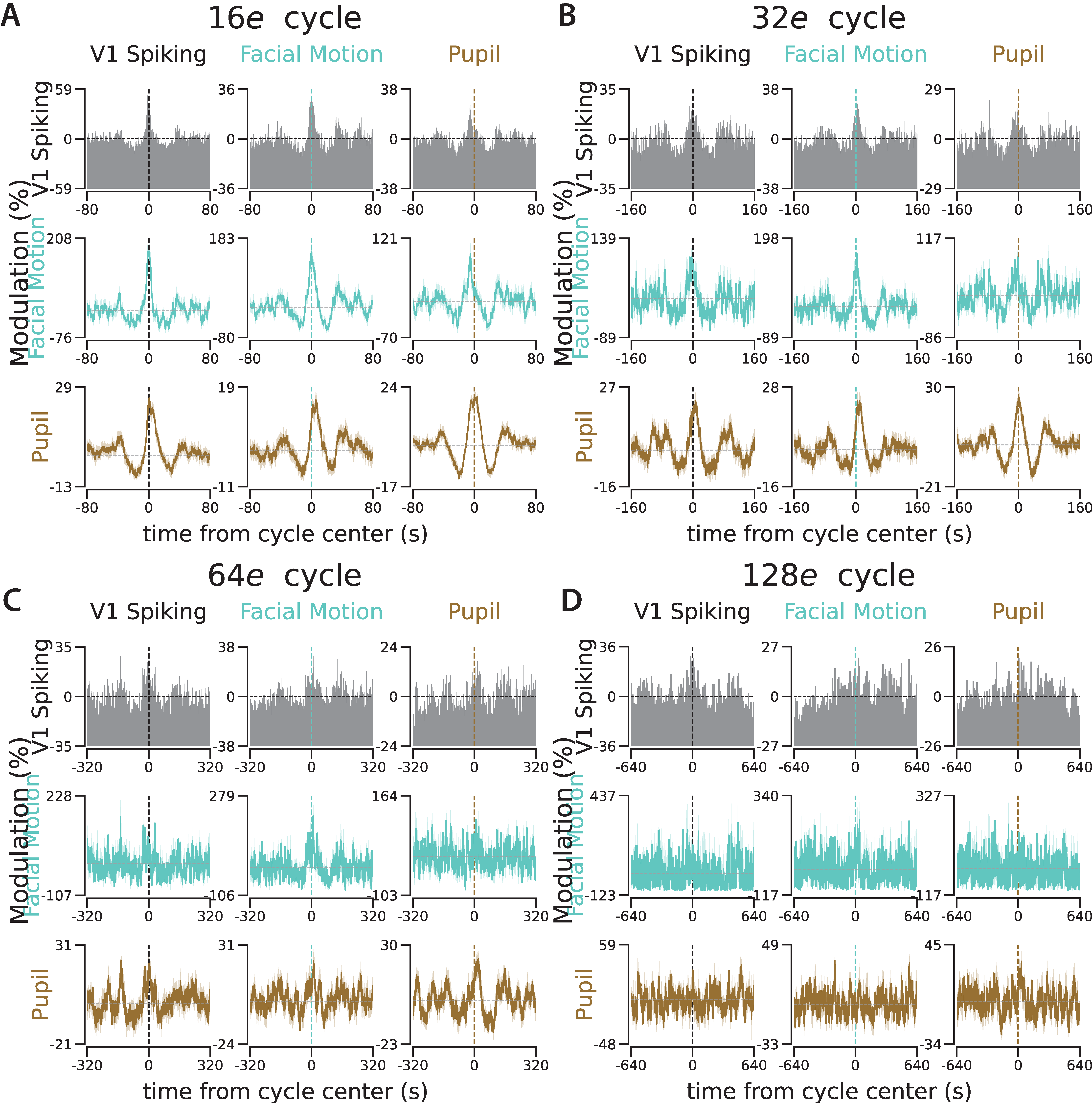

Supplement: S17 Fig — Similar to Fig 4B & 4E for four other slow cycles. (TIFF) [file pcbi.1013084.s017.tif]

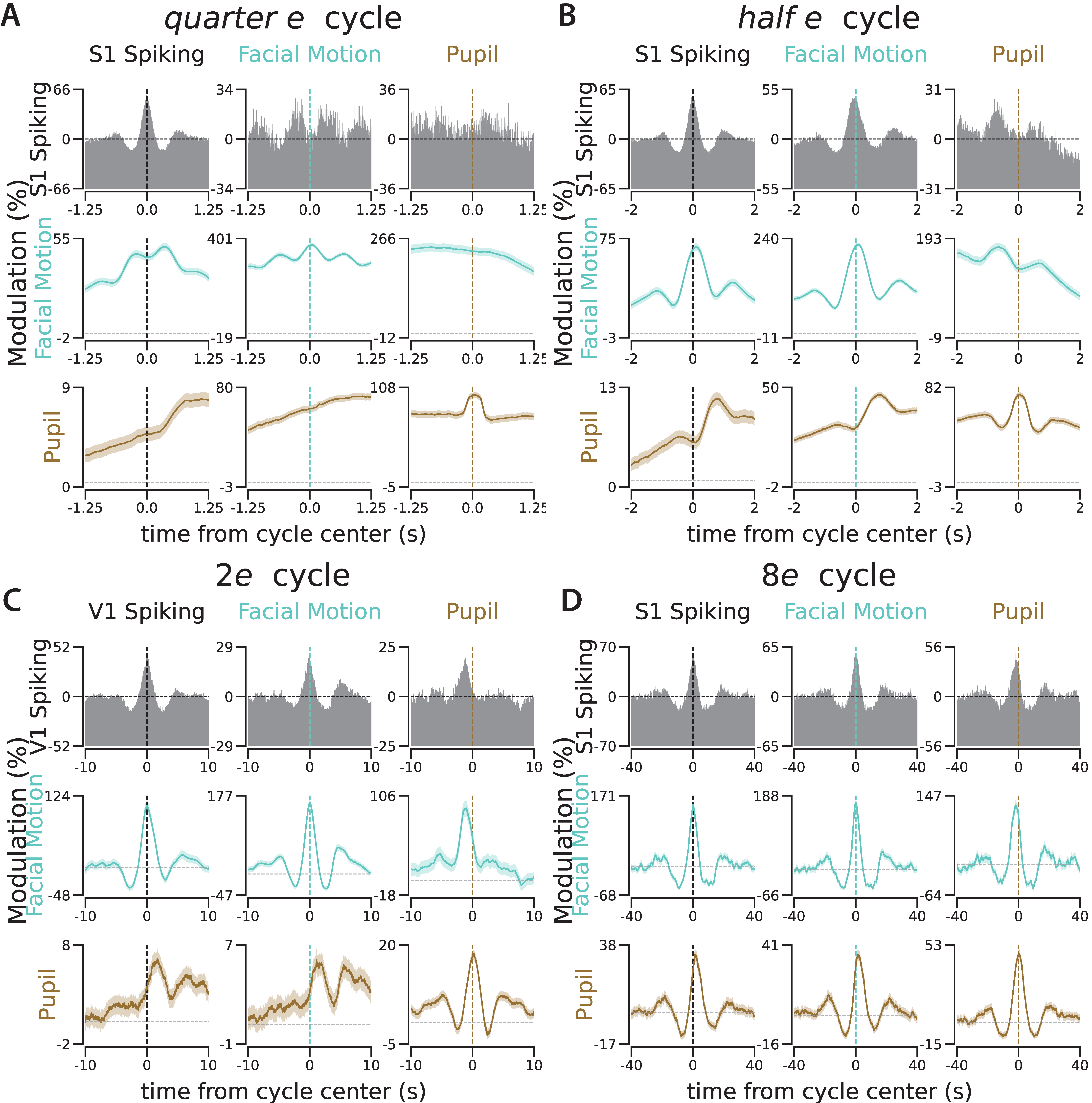

Supplement: S18 Fig — Similar to Fig 4C & 4F for four other slow cycles. (TIFF) [file pcbi.1013084.s018.tif]

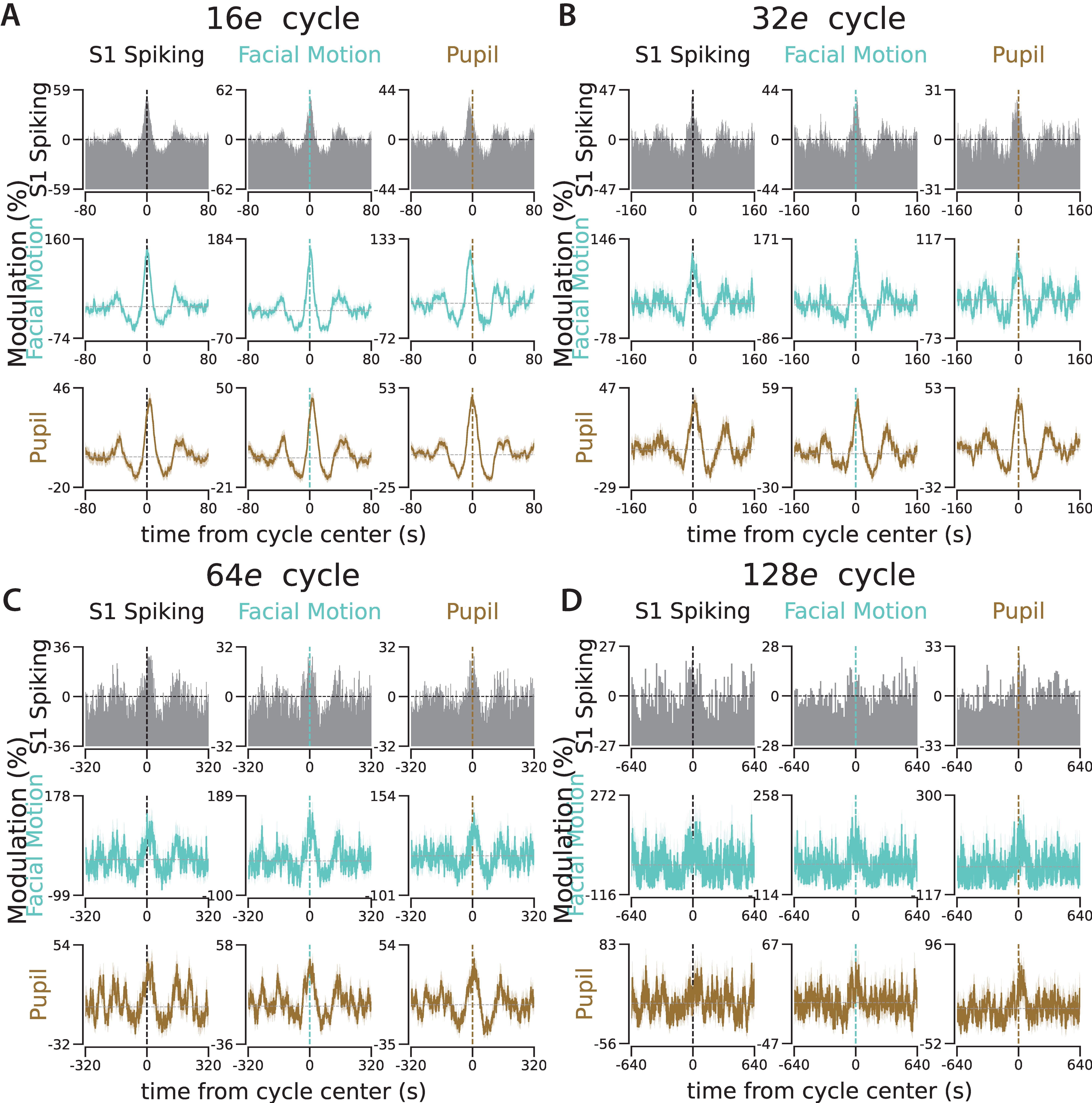

Supplement: S19 Fig — Similar to Fig 4C & 4F for four other slow cycles. (TIFF) [file pcbi.1013084.s019.tif]

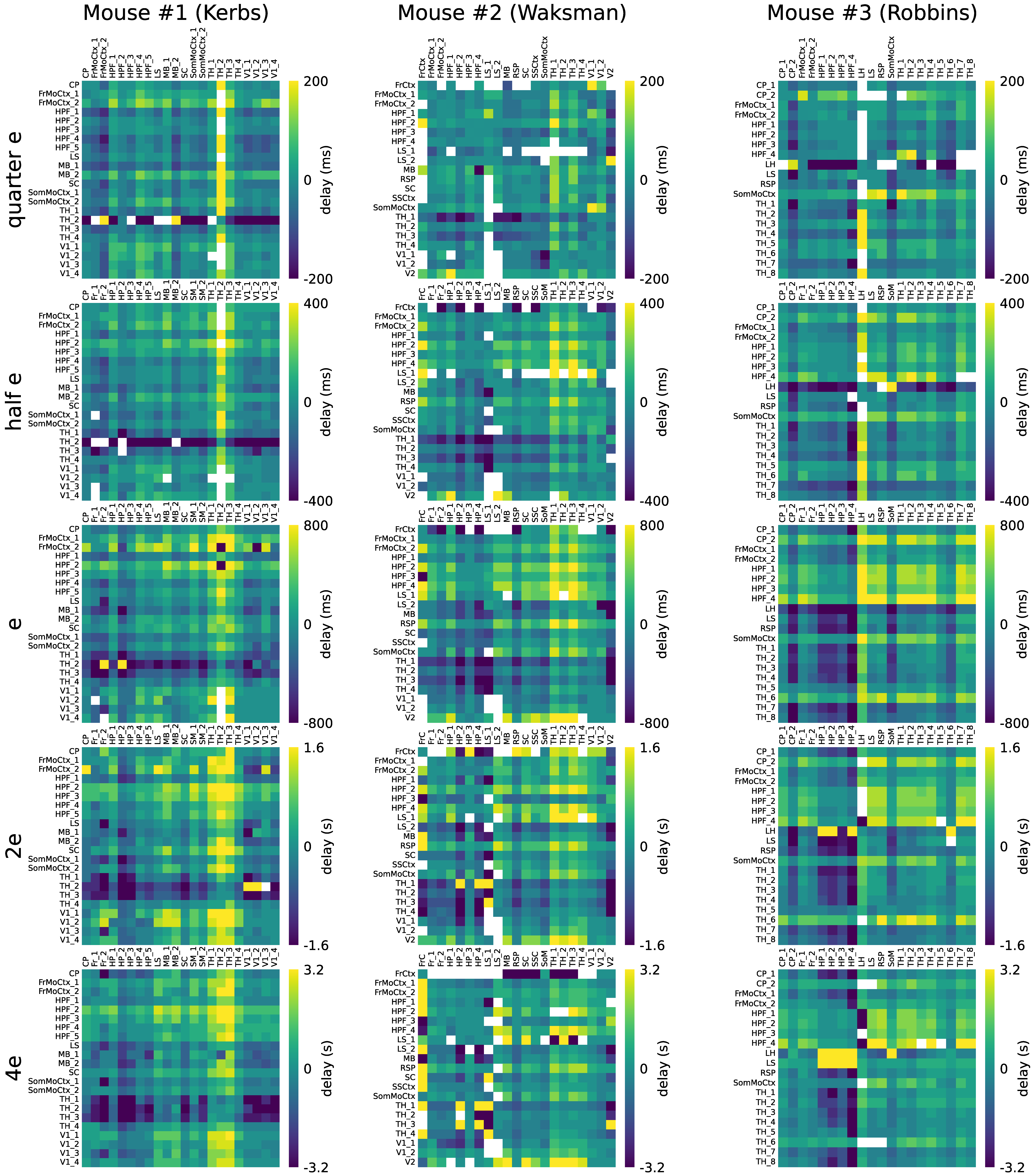

Supplement: S23 Fig — Similar to Fig 5C for quartere, halfe, e, 2e, and 4e cycles across all regions in 3 animals. If the pattern of correlated activity between two regions is not significant (see Methods), then the corresponding cell in the delay matrix is left blank. Patterns of correlated activity between regions, from which these delays were derived, can be found at [61]. (TIFF) [file pcbi.1013084.s023.tif]

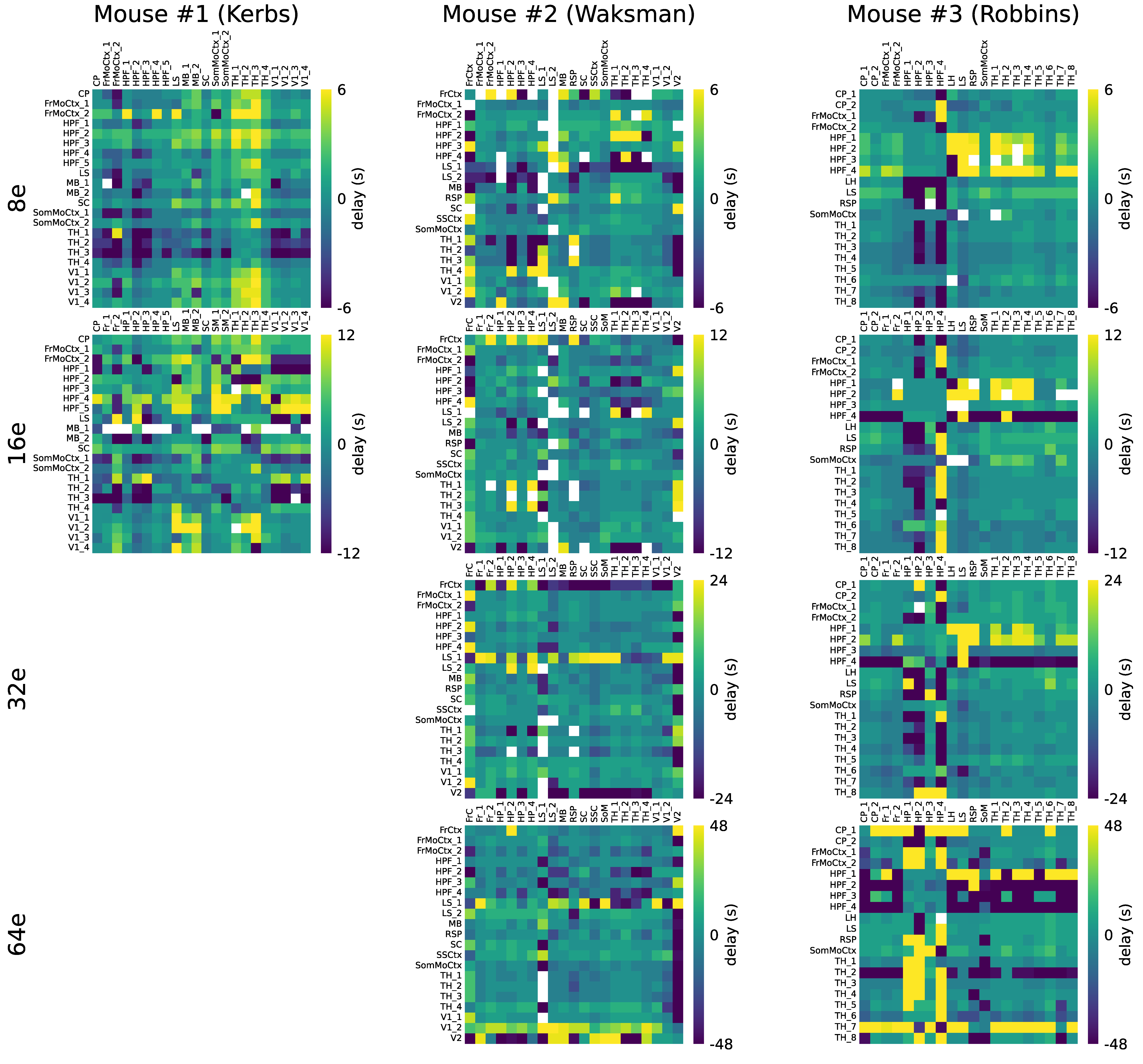

Supplement: S24 Fig — Similar to Fig 5C for 8e, 16e, 32e, and 64e cycles across all regions in 3 animals. If the pattern of correlated activity between two regions is not significant (see Methods), then the corresponding cell in the delay matrix is left blank. Patterns of correlated activity between regions, from which these delays were derived, can be found at [61]. (TIFF) [file pcbi.1013084.s024.tif]
