# Supplementary material for: Isolating single cycles of neural oscillations in population spiking
Source: PLoS Comput Biol. 2025 Jun 4;21(6):e1013084. doi: 10.1371/journal.pcbi.1013084 (PMC12136316; doi:10.1371/journal.pcbi.1013084)
Supplement: S1 Note — (PDF) [file pcbi.1013084.s025.pdf]

Supplementary Notes

Cycles and Power Spectrum Schematics in Figure 1.

To generate the panels A and B in Figure 1, we used the function $f(t)$, which is a solution for the motion equation of a harmonic oscillator[1]:

$$y''(x) + y(x) = 0, \qquad x(t) = 2\pi \log_2 \tfrac{t}{e}, \qquad f(t) = y(x(t))$$

$$t^2 f''(t) + t f'(t) + \left(\tfrac{2\pi}{\log 2}\right)^2 f(t) = 0, \qquad \boldsymbol{f(t) = -\cos(2\pi \log_2 \tfrac{t}{e})}$$

[1] https://math.stackexchange.com/questions/139881/how-to-construct-and-oscillation-with-exponentially-gro
