# Supplementary material for: Isolating single cycles of neural oscillations in population spiking
Source: PLoS Comput Biol. 2025 Jun 4;21(6):e1013084. doi: 10.1371/journal.pcbi.1013084 (PMC12136316; doi:10.1371/journal.pcbi.1013084)
Supplement: Attachment — Submitted filename: Response to Reviewers.pdf [file pcbi.1013084.s028.pdf]

Dear Editorial Committee,

Thank you for the opportunity to revise our manuscript and for the constructive feedback from the reviewers. We appreciate their thorough assessment and helpful suggestions for improving the manuscript.

Both reviewers highlighted the need to demonstrate our method's advantages over existing signal processing approaches. In response, we have added comparisons between our LSTM-based method and other approaches (spectral analysis and bycycle method) using synthetic data (Figure S1-2).

The reviewers also raised important points about the biological interpretation of our results, which we addressed in the point-by-point responses below. We have also clarified the relationship between LFP and spiking oscillations, acknowledging cases where dissociations were reported between LFP and spiking oscillations.

Additionally, we have:

- Improved the clarity of definitions and terminology throughout
- Added relevant citations and context
- Enhanced figures based on reviewer suggestions

The detailed point-by-point responses to the reviewers' comments follow below.

Reviewer #1:
This study presents a LSTM-based cycle detection method and applies it on detecting the gama cycle to align gamma cycles with trials and on calculating cycle-wise correlations across multiple brain regions and across time scales. Overall, the method seems novel. But some major flaws should be addressed before being further considered for publishing.

The reviewer thinks the method must be solidly validated before going further to use it on analyzing neuronal oscillations.

We thank the reviewer for the helpful comments and suggestions. To validate our method against other methods on synthetic data, we have added comparisons with the spectral analysis and another time-domain cycle detection method, namely the bycycle method, as shown in Figure S1-2. Please find our descriptions of how we addressed the points below.

1. Is the method of synthesizing training data and test data with ground truth valid enough to represent neuronal oscillations? In other words, how to guarantee the trained LSTM-based algorithms are generalizable enough to be applied on real neuronal signals?

This is a valid point; it is essential to demonstrate that the synthesized signal used for the training, aligns with the characteristics of the biological signal. To address this concern, we have chosen the variables for constructing the synthesized signal so that the power spectrum of the

synthesized signal matches the power spectrum of the population firing rate of the neural recordings. The variables we changed to match the population firing rate are the variability in the duration of each instance of the cycles (basis functions), the variability in the delay between the consecutive cycles of the same type, and the noise level (Fig. S1-1). To further validate the results of the cycle detection with this method, we showed that the detected cycles in the spiking activity reflect similar cyclic activity in the LFP (Fig. 1F), (LFP has not been used by our method for the cycle detection). We also showed that the detected cycles with our method reflect the cross-frequency coupling, a well-established neural phenomenon. To better describe how the synthesized signal used for the training is built, we have added the typeset of the formula for the synthesized signal to the method section:

$$s_{\text{train}}(t) = \text{Normalize}\left(\sum_{i=1}^{7}\sum_{j=1}^{N_i} \alpha_{ij} c_{ij}(t - t_{ij})\right) + \eta(t)$$

where:

- $c_{ij}(t)$: The Gaussian function representing the j-th instance of the i-th cycle type, defined as:

$$c_{ij}(t) = \exp\left(-\frac{1}{2}\left(\frac{t}{\sigma_{ij}}\right)^2\right)$$

- σ_{ij}: The jittered standard deviation (width) for the j-th instance of the i-th cycle type, defined as:

$$\sigma_{ij} = \sigma_i + \Delta\sigma_{ij}, \quad \Delta\sigma_{ij} \sim \mathcal{N}(0, 0.3 \cdot \sigma_i)$$

  where $\sigma_i = \frac{d_i}{5}$.
- d_i: The base duration of the i-th cycle type, given by:

$$d_i = 2^{i-4} \cdot d_4$$

  Here, d_4 is the duration of the target cycle.
- d_{ij}: The jittered duration of the j-th instance of the i-th cycle type, related to σ_{ij} as:

$$d_{ij} = 5 \cdot \sigma_{ij}$$

- t_{ij}: The center time of the j-th instance of the i-th cycle type, ensuring that instances of similar cycle types don't overlap:

$$t_{ij} = t_{i(j-1)} + d_{i(j-1)}$$

  t_{4j} time points are the targets of decoding.
- α_{ij}: A binary inclusion variable sampled as:

$$\alpha_{ij} \sim \text{Bernoulli}(p), \quad p = 0.6$$

- Normalize$(\cdot)$: The normalization function scales the synthesized signal to lie between 0 and 1:

$$\text{Normalize}(s) = \frac{s - \min(s)}{\max(s) - \min(s)}$$

- $\eta(t)$: Additive Gaussian noise sampled as:

$$\eta(t) \sim \mathcal{N}(0, \sigma^2), \quad \sigma = 0.05$$

2. How to determine the hyperparameters, e.g. the sliding window length, noise level, batch size, optimizer used for training, the data splitting protocol for validation. And how many cycles on average are included in a batch?

Thank you for giving us the opportunity to add the missing information to the method section:

“The LSTM network consisted of two layers of 200 units each. The input is sampled at 30 samples/(target cycle). The input to the network at each time point consists of 61 samples, including 30 samples before and 30 samples after the current time point. The output layer of the network consists of one dense unit (one sample corresponding to the center sample of the window provided as the input at each step) with linear activation that reflects the probability of the detection of the target cycle at each time point. 85% of the synthetic data was used for the training and 15% was used for testing until the convergence based on the accuracy. The batch size was set to 128. Root Mean Square Propagation (RMSprop) was used for the optimization and Mean Square Error (MSE) was used as the loss function. To introduce noise to the signal during training, a Gaussina_Noise layer (stddev: 0.05) was added immediately after the input layer. A synthesized signal with 3 million samples was used to train the network. The synthesized signal was generated using seven cycle types as basis functions. The duration of each cycle type doubled relative to the previous one (3 cycles shorter and three cycles longer than the target cycle for detection). Each type of cycle occurred with a 60\% chance. Different cycle types are randomly combined in all possible ways, while cycles of the same type do not overlap. The width of each cycle at each instance was randomly jittered by adding a value derived from a normal distribution with $\mu=0$ and σ set to 30\% of the cycle width. These parameters were chosen so that the spectral density of the synthesized signal matches the average spectral density of the population firing rate at 29 recording sites across the mice brain from two datasets (\Cref{fig:s1}A)”

3. What's the cost function of the algorithm?

We used an MSE loss function and have added the typeset of the loss function to the method section:

For training, the network output was set as a binary one-dimensional time series of the same length as the input. This output signal was set to zero everywhere except at the center of the target cycle.

$$y(t) = \begin{cases} 1, & \text{if } t = t_{4j} \text{ (center of the target cycle)} \\ 0, & \text{otherwise} \end{cases}$$

The sampling rate of the synthesized signal was set so that the target cycle was sampled 30 times per cycle. For predicting each output sample 61 samples from the input were provided to the network including 30 samples before and 30 samples after the target sample. A Mean Squared Error (MSE) loss function was used for the training:

$$\mathcal{L} = \frac{1}{T}\sum_{t=1}^{T}(y(t) - \hat{y}(t))^2$$

4. What is the LSTM-based method's benefit comparing with SOTA methods?

Other methods mainly use band-pass filtering, which has a limited capacity to handle the variability in the duration of the cycles and the variability in the delay between the consecutive cycles to estimate the center of the cycles and correctly align them. A supervised training using a synthesized signal provides the ability for the network to learn to handle these sources of

variabilities in noisy conditions. We added the analyses to compare our method with other methods as shown in Fig. S1-2. Please note the high False Alarm rates of these methods that reflect the mishandling of the longer periods without cycles.

Other questions on the following analysis are

*What is the purpose of the following analysis? To validate the algorithm against the known phenomena or to find some novel results with the new tool? If the former, the authors are suggested to add further comments on what are the similarities between the results this study finds and the previously known phenomena. If the latter, it is highly suggested to find a way to validate the generalizability of the algorithm on real-world signal**s.***

The results presented in the first figure (except the panel L) are to validate against the known phenomena (i.e. cyclic activity in the LFP and cross-frequency coupling). The results presented in the following figures show the types of applications for our method as well as the new knowledge that can be gained by having an accurate estimation of the time of single cycles in the spiking activity across different time scales (i.e. frequency bands): 1-better alignment of trials in response to sensory stimulation 2- time scale dependent correlated activity across brain regions (and also behavioral measures of arousal). We added the following texts to address these points:

"We trained the decoder using a synthesized signal, constructed with neural cycles as basis functions (\Cref{fig:fig1}B), and incorporated noise and variations in the width of each cycle instance to match the spectral characteristics of the biological signal (\Cref{fig:s1,fig:fig1}B-C, \nameref{sec:Methods}). Using the trained decoder, we then identified the timing of individual cycles underlying the fluctuations in the population spike counts during extracellular recording experiments. To test the validity of the detected cycles, we showed that the average LFP around these cycles, detected in population spiking, reflects similar cyclic patterns. We also demonstrated the known neural cross-frequency couplings using the isolated cycles in the population spiking."

Reviewer 2
Summary
The authors propose a neural network-based approach to detect single cycles of oscillatory activity from population spiking data. The use of an LSTM architecture pre-trained with synthetic data is noteworthy, and demonstrates a novel application of decoding algorithms for posing scientific questions. There also seems to be a clear relationship between "single cycles" detected in the spiking, and the LFP/physiologic signals aligned to these cycles. The authors also provide intriguing interpretations on the computational role of correlations between population spiking cycles and different brain regions/recording modalities.

The major shortcoming of this manuscript is that it is applying a new neural network-based method for something that has standard alternatives in signal processing (e.g. using a

spectrogram to detect transient increases in spectral power). While there is some brief justification on why transient oscillation/cycles may be difficult to detect, this needs to be shown explicitly rather than assumed. Further, this method is being used to characterize neural signals in a unique manner (oscillations of sum total population spiking), and interpret the biological significance. If the goal of this manuscript is to argue for the use of this method, then there needs to be concrete comparisons to existing tools, and a commentary on their strengths and weaknesses. If the goal is to characterize the significance of oscillations in population spiking, the same advice applies, as it's difficult to assess if this is a quirk of the tool (and the assumptions of the synthetic training data), or a fundamental and biologically significant signal.

We thank the reviewer for elaborating on the shortcomings of the manuscript and for the constructive suggestions. We now included the comparisons of this method with the standard spectral analyses and another cycle detection method in Figure S1-2 as suggested by the reviewer. We have revised the text to provide a more detailed explanation of how changes in the combined population spiking could reflect a neural phenomenon similar to that observed in the LFP. Additionally, we have included citations to other studies that have utilized a similar measure. We also noted the studies that point to the dissociation between the oscillations in LFP and spiking as pointed out by the reviewer (Lines 54-65 in the revision without track change). You can find more details about the corresponding changes to these major issues in our responses to the specific comments below.

It should be noted that the writing and figures are of high quality. However, the manuscript would greatly benefit from reworking the definitions/descriptions of the LSTM method to add clarity. Given the current state of the manuscript, I recommend to accept with major revisions.

Main Comments

In all sections of the manuscript, it needs to be made obvious that 1) "population spiking" means the sum of all neuron firing rates (this term is ambiguous with respect to it being a multivariate or univariate signal), and 2) the oscillations detected are specific to this population-level spiking, and is not directly applied to LFP features. Throughout the introduction I was left thinking that this method inputs spiking data to the LSTM, and outputs predictions on LFP oscillations. The discussion results do a better job of making this distinction in some places, but in many places these details have to be inferred as the language used leaves room for ambiguity. A very specific example is L55 in the introduction which reads as very vague, I encourage you to add explicit definitions for these terms.

We have now rewritten paragraph two in the introduction to explicitly indicate that the population spike rate on which we run the decoding is built by pooling spikes from individual single units in a local network. We have also changed the text in other parts of the manuscript to make sure that the meaning of the population spiking is clearly communicated.

"We hypothesize that individual cycles of rhythmic activity in neuronal spiking can be identified by analyzing changes in the local population spike count. To achieve this we aggregated spikes from all single units within a densely recorded neuronal population and measured the population

spike rate over time, treating it as a univariate signal \citep{Okun2019DistinctTimescales,Okun2015DiverseCortex}. In densely recorded extracellular activity, where tens of neurons are recorded simultaneously within a local network, variations in the rate of population spikes reflect shared fluctuations in sub-threshold activity across the network. Sub-threshold activity has also been shown to follow the LFP \citep{Haider2016MillisecondCortex, Perrenoud2016MembraneMice}. Taken together, these observations suggest that patterns of cyclic neural activity can be identified by analyzing the pooled spiking rate of the recorded population. However, it should be noted that the rhythmicity in LFP and spiking activity are not always correlated, for example, beta-band activity in the primate motor cortex is dissociated between LFP and neuronal spiking \citep{Rule2017DissociationCortex,Confais2020IsCortex}.

To test this hypothesis, we employed Long Short Term Memory (LSTM) networks as decoders to isolate individual cycles of various neural rhythms in the spiking of neurons. We accomplished this by analyzing the variations in the population spike rate, when pooled together spikes from all the recorded neurons in a local network, in the time domain. LSTM is a powerful tool for analyzing time-series data, capable of considering the history of the signal to identify learned events \citep{Hochreiter1997LongMemory}, which makes it well-suited for our investigation. We trained the decoder using a synthesized signal, constructed with neural cycles as basis functions (\Cref{fig:fig1}B), and incorporated noise and variations in the width of each cycle instance to match the spectral characteristics of the biological signal (\Cref{fig:s1,fig:fig1}B-C, \nameref{sec:Methods}). Using the trained decoder, we then identified the timing of individual cycles underlying the fluctuations in the population spike counts during extracellular recording experiments \citep{Rule2017DissociationCortex,Confais2020IsCortex}."

While it is certainly true that spiking in the local recorded population can produce a measurable change in the LFP, the reverse is not necessarily true as (e.g. subthreshold oscillations). Even in the suprathreshold case, motor cortical beta oscillations are a great example where there is a measurable change in local spiking, but it is a global reduction that is not phase locked to the oscillation. This is a really important caveat to mention in the introduction/discussion, as the paper seems to establish an equivalency between these signals. References specific to beta oscillations are below:

Rule, M. E., Vargas-Irwin, C. E., Donoghue, J. P., & Truccolo, W. (2017). Dissociation between sustained single-neuron spiking and transient β-LFP oscillations in primate motor cortex. Journal of Neurophysiology, 117(4), 1524-1543.

Confais, J., Malfait, N., Brochier, T., Riehle, A., & Kilavik, B. E. (2020). Is there an intrinsic relationship between LFP beta oscillation amplitude and firing rate of individual neurons in macaque motor cortex?. Cerebral Cortex Communications, 1(1), tgaa017.

Thank you for pointing out the dissociation of the LFP and spiking during the beta band activity in the motor cortex. We have added the following text to the introduction and discussion

sections to mention these studies indicating that the neural oscillations reflected in spiking and LFP are not intrinsically (co)related. We also changed the abstract accordingly (please see the changed abstract in response to the points regarding the abstract).

Introduction:
“Taken together, these observations suggest that patterns of cyclic neural activity can be identified by analyzing the pooled spiking rate of the recorded population. However, it should be noted that the rhythmicity in LFP and spiking activity are not always correlated, for example, beta-band activity in the primate motor cortex is dissociated between LFP and neuronal spiking \citep{Rule2017DissociationCortex,Confais2020IsCortex}.”

Discussion:
“To shed light on this controversy, it is helpful to consider a related question: How much of the rhythmicity observed in the LFP can be captured solely from spiking activity? The LFP reflects population-level activity, capturing rhythmicity when synchronized rhythmic input is delivered to the network by axons or when neighboring neurons share synchronized subthreshold oscillations. However, if the rhythmic signals are not synchronized, their contributions cancel out in the effective extracellular fields recorded by the LFP. In contrast, the spiking activity of individual neurons has limited power to detect such rhythmicity compared to the LFP. By aggregating the spikes from nearby cells, however, we can create a population measure of spiking activity. The population firing rate derived in this way, as proposed by \citep{Okun2019DistinctTimescales,Okun2015DiverseCortex}, reflects shared variations in the membrane potentials of nearby neurons and offers a spiking-based measure of population activity. This measure can capture network cyclic activity in a manner comparable to the LFP. However, it is important to note that the relationship between cyclic activity in the LFP and neuronal spiking is not inherently intrinsic. Cases of dissociation between LFP oscillations and neuronal spiking have been observed. For example, beta-band activity in the primate motor cortex shows such dissociation \citep{Rule2017DissociationCortex,Confais2020IsCortex}.”

Spectral analysis of firing rate summed across every neuron is not very common in computational neuroscience, especially applied to neuropixel data. It would be highly beneficial to add references that use this technique, up/down states during sleep come to mind. Otherwise it will be important to outline precisely why you think such a signal is important as it relates to neural oscillations.

It is correct that combining spikes from different neurons is not a common approach. To address this, we have revised the text to include citations to other studies that have utilized the combined population spiking rate. Additionally, we have outlined the potential relation between the combined population firing rate and shared variations in the subthreshold activity within the local neuronal population.

“We hypothesize that individual cycles of rhythmic activity in neuronal spiking can be identified by analyzing changes in the local population spike count. To achieve this we aggregated spikes from all single units within a densely recorded neuronal population and measured the population

spike rate over time, treating it as a univariate signal \citep{Okun2019DistinctTimescales,Okun2015DiverseCortex}. In densely recorded extracellular activity, where tens of neurons are recorded simultaneously within a local network, variations in the rate of population spikes reflect shared fluctuations in sub-threshold activity across the network. Sub-threshold activity has also been shown to follow the LFP \citep{Haider2016MillisecondCortex, Perrenoud2016MembraneMice}. Taken together, these observations suggest that patterns of cyclic neural activity can be identified by analyzing the pooled spiking rate of the recorded population."

It is a shame that there isn't analysis in layer-specific differences in population spiking, as there is a rich literature on the unique contributions of deep/superficial layers to slow/fast oscillations. I'll leave it to your discretion if this is within the scope of your paper (see my comment in the summary on focussing on methods vs. biological significance). If the focus of the paper is the biological significance, then a layer-specific analysis would provide much more compelling predictions on the biophysical mechanism relating population spiking to LFP oscillations. I don't think single unit spike sorting would be necessary as it comes with many pitfalls. However, as you reference in the discussion, a major advantage of this technique compared to LFP's is isolating the spatial location of neurons being summed over (of course I will challenge this statement as current source density analysis precisely aims to spatially localize LFP contributions).

This is indeed a very interesting analysis that can be done with this tool, the reason that we have not included the results of this analysis in this manuscript is that the number of spikes that are recorded in the superficial cells is significantly lower than the spikes recorded in the deeper layers. And since the length of these recordings is limited (~ 2 hours), the number of reliably detected cycles in the superficial layers is not enough to examine the intra-layer cycle relations. As a next step, we are planning to perform long (> 10 hours) chronic recordings across different behavioral states (quiet wakefulness, movement, REM, and SWS sleep) and collect a dataset tailored for this analysis.

There is a fundamental signal processing question that should be addressed: is an LSTM the right tool to detect transient oscillations? You cite cycle by cycle analysis (https://github.com/bycycle-tools/bycycle, Cole and Voytek 2019), why not apply this technique to the population spike rate as well? Other similar techniques with associated neuroscience publications include "burst detection" (https://github.com/danclab/burst_detection) and "spectral event analysis" (https://github.com/jonescompneurolab/SpectralEvents), both of which characterize transients in the time-frequency spectrogram. The simplest approach would be tracking threshold crossings in the envelope of a band-passed signal. A direct comparison to at least one existing approach would be highly informative and really strengthen the results of this paper. I certainly support the use of deep learning in science, but when analytic alternatives are available these neural network based approaches need to be benchmarked to build trust in the method. I'd be much more convinced if the LSTM outperforms standard methods in terms of AUC when applied to your synthetic dataset.

Thank you for this suggestion. We have now included in Fig S1-2 the result of the comparison with “bycycle” (Cole and Voytek 2019) as well as the threshold crossing approach on the bandpass filtered signal suggested by the reviewer. Please note that bycycle doesn’t provide the output result of peak detection with varying thresholds, and therefore the generated Hit vs FA curve for this method has a limited range. While powerful for the detection of bursts of rhythmic activities, the high FA rate of these methods in detecting individual cycles points to the limited capacity of the spectral analysis in handling the variations in the width of the cycles as well as the delay between consecutive cycles.

The github repository attached does not seem to include code to generate the synthetic data, or code for training the neural network. While it is clear that a lot of effort went into making this a usable python package, this repository does not allow me to reproduce the results in this manuscript. Even if the training code is not in a clean state, it is important to include this information to properly validate the claims in the manuscript.

We shared the code for producing the synthesized signal and the training here
https://www.dropbox.com/scl/fo/xpgdys1nysatanc407yho/ADfM9AOqTHejyckDS1b-2bQ?rlkey=62sn6tb8bln9m9x3hl48wv3e5&st=tu9zyhht&dl=0
We will post this as a standalone github repository publicly as soon as possible after cleaning the code to make it more straightforward for people to use.

Abstract

It is not a given that oscillations in EEG, LFP, and spiking have an “intrinsic” relationship. They can certainly correlate, but it is not universally true that neural oscillations are detectable on all 3 modalities given the same underlying mechanism. I think you should rewrite this to assert that oscillations in population spike rates are present, likely to be biologically significant, and should be treated as a distinct form of neural activity in its own right. You can even state that there is a fundamental gap in understanding 1) how spiking oscillations relate to field potential oscillations, 2) how spiking oscillations are correlated across brain regions, and 3) how spiking oscillations are correlated to physiological signals and behavior.

Those are excellent points and we are very grateful for your input. We rewrote the text as below considering these points, leading to a much better abstract.

It is strange to state that spikes “estimate the network’s activity over time”. It is a small sample of the entire network. Do you mean this is an estimate of the global network activity?

Thank you for pointing this out and providing an opportunity to clarify. By “network activity” we were referring to the shared subthreshold activity within a very local network. However, since the neural signals can exhibit various spatiotemporal components depending on the size of the network being considered, we have revised this part of the text for greater clarity.

“Neural oscillations are prominent features of brain activity, characterized by frequency-specific power changes in electroencephalograms (EEG) and local field potentials (LFP). These oscillations also appear as rhythmic coherence across brain regions. While their identification has traditionally relied on EEG and LFP, oscillations are also present in neuronal spiking. However, several questions remain unanswered: How do spiking oscillations relate to field potential oscillations? How are spiking oscillations correlated across brain regions? And how are they connected to other physiological and behavioral measures? In this study, we explore the potential to detect individual cycles of neural rhythms solely through neuronal spiking activity, leveraging recent advancements in the densely recording of large neuronal populations within local networks. The pooled spiking rate of many neurons within a local population reflects shared variations in the membrane potentials of nearby neurons, allowing us to identify cyclic patterns. To achieve this, we utilize a Long Short Term Memory (LSTM) network, pre-trained on synthetic data, to effectively isolate and align individual cycles of neural oscillations from densely recorded neuronal populations. We applied this approach to robustly isolate specific neural cycles across various brain regions in mice, covering a broad range of timescales, from gamma rhythms to ultra-slow rhythms lasting up to hundreds of seconds. These ultra-slow rhythms, often excluded from the LFP, were also detected in behavioral measures of arousal, such as pupil size and mouse facial motion. Interestingly, these rhythms showed delayed coherence with corresponding rhythms in the population spiking activity. Using these isolated neural cycles, we addressed two key questions: 1) Precise Alignment of Sensory Trials: By isolating gamma cycles driven by sensory input, we achieved a more accurate trial alignment in the sensory stimulation experiments conducted in the primary visual cortex (V1) of mice. This alignment accounts for biological variability in sensory signal transmission times from the retina to V1 across trials, enabling a clearer understanding of neural dynamics in response to sensory stimuli. 2) Cross-Region Correlations at Different Timescales: We used the distinct spiking cycles in simultaneously recorded brain regions to examine the correlation of spiking across brain regions, separately for different timescales. Our findings revealed that the delays in population spiking between brain regions vary depending on the brain regions involved and the timescale of the oscillations. This work demonstrates the utility of population spiking activity for isolating neural rhythms, providing insights into oscillatory dynamics across brain regions and their relationship to physiological and behavioral measures.”

<u>Introduction</u>

Numerous researchers have developed methods to characterize the relationship between spiking and field potentials. Spike-field coherence is the most apparent method that is absent from the introduction/discussion. It will be important to add a paragraph outlining the commonly used methodology for relating spikes to field potentials, and precisely how your approach is unique from these methods.

We changed the introduction as below to include the spike-field coherence as the analysis method that reveals the cyclic activities shared between the spikes and field potentials and discussed it in the context of our method:

“Individual neurons tend to fire spikes during specific phases of these rhythms \citep{Pesaran2002TemporalCortex, Sirota2008EntrainmentRhythm,Jacobs2007BrainHumans,Watrous2018Phase-tunedGoals} as revealed by methods that measure the coherency between the spiking activity and LFP \citep{Vinck2012ImprovedPotential,Davis2023Spike-phaseCortex}. This suggests that the probability of neuronal spiking is modulated by the same recurring temporal motifs—namely, cycles (\Cref{fig:fig1}B), which underlie the spectral changes observed in LFP. The duration of these cycles is linked to their respective frequency bands (\Cref{fig:fig1}B)...

We hypothesize that individual cycles of rhythmic activity in neuronal spiking can be identified by analyzing changes in the local population spike count. To achieve this we aggregated spikes from all single units within a densely recorded neuronal population and measured the population spike rate over time, treating it as a univariate signal \citep{Okun2019DistinctTimescales,Okun2015DiverseCortex}.”

The motivation for identifying “single cycles” is a bit vague and could be better stated. As it currently reads, this could be referring to either 1) detection of oscillatory bursts/events, or 2) accurate phase estimation in continuous rhythms (or perhaps both?). There is a growing body of literature emphasizing the importance of characterizing oscillations as bursts as opposed to rhythms (cycle by cycle analysis being one example that you cite) but this discussion is absent in the manuscript. It would help greatly to clarify what framework you’re working from, and add a few words on why treating neural oscillations as bursts or steady oscillations is important from a biological perspective. The discussion broaches this topic in more detail, but it would be useful to reference this work with 1-2 sentences in the introduction.

You are correct - identifying individual cycles will facilitate both the detection of oscillatory bursts and the accurate estimation and alignment of their phases. This, in turn, will enable the study of their computational role within neuronal circuits and their correlations with behavioral outcomes. We have incorporated this explanation in the introduction:

“Spectral analysis of neural signals, especially of LFP, has been the primary approach for linking rhythmic activities to various functions of the nervous system. Additionally, time-domain methods have been developed to detect repeating cycles in LFP. \citep{Cole2019Cycle-by-cycleOscillations,Perrenoud2022FlexibleEvents}. However, the extent to which spiking activity contains information about the timing of individual cycles remains unclear. Identifying individual cycles as discrete neural events offers improved temporal resolution, enabling more precise alignment of these cycles and improved detection of their oscillatory bursts \citep{Cole2019Cycle-by-cycleOscillations, Szul2023DiverseDynamics}. This enhanced temporal resolution is essential for investigating the computational roles of these cyclic events within neuronal circuits and their correlations with behavior.”

<u>Results</u>
Section 2.1

To better link these single cycles to a neural mechanism, it'd be highly beneficial to include a current source density (CSD) plot either in the main results, or as a supplemental figure. You make reference in L106 to some sort of propagation across layers. With a CSD plot you'd have a much more mechanistic description of this phenomenon in terms of current sinks/sources in different layers.

Thank you for the suggestion, we added the CSD plots to figure 1 as panel G.

Section 2.2

The results here are interesting, but I feel a direct comparison to a simpler technique is necessary. A very naive approach would be to apply a peak finder to the population spiking signal, and apply the jitter correction/reordering to any peaks above a certain threshold. If you compare these techniques side by side, does your LSTM outperform the peak finder in terms of some alignment quality metric?

To address this point, we included the results of trial reordering and jitter correction based on population firing rate peaks in each trial in Fig.S2-1. This analysis shows that while using peak timing for reordering and jitter correction can partially capture gamma cycle dynamics, it is less effective at accurately aligning trials or distinguishing consecutive cycles, as consecutive cycles do not always appear as distinct peaks. We added the following text to the corresponding result section to highlight this analysis:

"Reordering trials based on the timing of population firing rate peaks after the stimulus onset reveals gamma cycles of activity (\Cref{fig:s1_2}). However, this method provides less accurate trial alignment and less precise differentiation between adjacent cycles compared to using the timing of individual cycles for jitter correction."

Figures 2I-K are not described in the text, what is the motivation behind these analyses?

Thank you for highlighting this point. We added the description regarding these analyses to this section of the results:

"Behavioral state has been shown to modulate sensory-evoked responses \citep{Niell2010ModulationCortex}, as reflected in the locomotion, pupil size changes \citep{Vink}, and the mice facial motion. Among these, facial motion is more temporally synchronized with network activity changes than pupil size \Citep{Sabri2024VasoactiveActivity} and is better at capturing subtle changes during non-locomotion periods. We observed that facial motion correlates with baseline population spiking rate across trials (\Cref{fig:fig2}I) and it also correlates with the strength and timing of upcoming sensory-driven gamma cycles (\Cref{fig:fig2}J-K). Specifically, gamma cycles in high-arousal trials are stronger and arrive at the visual cortex faster."

In L149 it would be useful to add references to support the "discrete packets" hypothesis. The references in the discussion are great so just add them here.

The references were added here.

“This suggests that sensory information is passed to the visual cortex in discrete packets \citep{Luczak2013,Luczak2015Packet-basedCortex}.”

Section 2.3

This is a cool analysis, but terms like “cycle type” and “time scales” make it hard to understand what’s being shown. This could be mitigated by very explicitly defining what you mean by “cycle type” (i.e. different frequency bands that are prominent in neural activity).

I’m still not entirely sure if “different time scales” refers to different cycle types, or the delay between regions. Again explicit definitions would help a lot.

Thank you for pointing out this problem, we added the correspondence between the time scales and the frequency bands explicitly and also added reference to the schematic in Fig. 1A to further clarify.

“Coherent neural activity, defined by synchronized rhythmic patterns, is widely regarded as a measure of information transfer between brain regions \citep{Fries2015RhythmsCoherence}. This coherence, typically measured using LFPs recorded in different brain regions, has been observed across various frequency bands and species \citep{Brincat2021InterhemisphericMemories,Schoffelen2005NeuronalInteraction,Schneider2021APower} and has been linked to numerous cognitive functions \citep{Buschman2007Top-downCortices,Bastos2015VisualChannels,Michalareas2016Alpha-BetaAreas}. Our goal was to investigate intra-areal coherence in the spiking activity across different time scales, corresponding to various frequency bands. To achieve this, we applied cycle isolation to a dataset containing simultaneous recordings from multiple brain regions \citep{Steinmetz2019Eight-probeBehaviors}. For each brain region, we identified cycles in the population spiking activity at different time scales (representing various frequency bands, see \Cref{fig:fig1}A}). Next, we calculated the average spiking patterns in other regions relative to the cycles detected in the reference region, analyzing each cycle type (corresponding to different frequency bands, see \Cref{fig:fig1}A) separately.”

The delay-matrices in Figure 3C-F are not described in the text, either remove these panels or give them a complete description.

We added the below text to describe the delay matrices:

“To determine the delay between two regions for a specific cycle type (i.e. frequency band), we first checked whether the pattern of correlated spiking between the regions was significantly different from chance. If so, we estimated the delay as the time corresponding to the peak of the distribution of their correlated spiking, derived using the isolated cycles. To summarize the correlated spiking patterns across all region pairs, we constructed delay matrices for each cycle

type (\Cref{fig:fig3}C-F show example delay matrices between specific regions in two animals for theta and delta bands, while \Cref{fig:s13} presents the full delay matrices of all cycle types)."

Section 2.4

L175-L177: High pass filtering of LFP's is a signal processing choice that is explicitly used to eliminate physiological signals like breathing and motion artifacts. It's misleading to present population spiking as a better readout of ultra-slow signals when you can simply change the filter of LFP's. Do you have any reason to think they might be a better read-out of ultra-slow oscillations? Of course I'll note my earlier concern that oscillations in population spiking are not necessarily correlated with the same frequencies of oscillation in LFP, a caveat worth denoting in the introduction.

By ultra-slow cycles we mean <0.1Hz frequency bands that are filtered out during the recording because of the hardware limitations in ordinary recording setups (although in dedicated works, researchers have used specialized recording setups, called DC recording, to capture these very slow dynamics, such as Norton and Jewett (1965) DOI: 10.1016/0013-4694(65)90162-8). We edited the text to clear this point.

"By isolating cycles in neuronal spiking, we can also study slower rhythmic activities (< 0.1 Hz) typically filtered out in the LFP because of the limitations of recording hardware."

L184: Is "the value two for the ratio…" a typo?

The ideas of relating oscillations to euler's number and the golden mean are quite intriguing. It would be much more convincing to show that these cycle widths actually correspond to something tangible in the frequency spectrum of the data rather than treating it as an a priori assumption (perhaps a spectrogram could be used to at least show transient increases in these ultra slow bands?). Given the power spectrum shown in supplemental figure S1A, I'm a bit suspicious that you could pick any cycle width and get something that looks reasonable. You touch on this problem in L259 of the discussion section, but the logic is somewhat strange given that in the results you argue these ratios are important based off of well observed peaks in the power spectrum of neural signals, but in the text you state that many things are missed when you look for peaks in the power spectrum. It just seems like a shaky justification for choosing these values, and highlighting these limitations in the discussion (or briefly in the results) will be useful for interpreting the significance of these findings.

We completely agree with the reviewer on this point. We have mentioned in the discussion the need for unsupervised identification of the basis functions as the next immediate step for this method (and similar methods). We used this specific number as the ratio between adjacent bands as a starting point for less explored slow oscillations since it matches the relation between established faster bands and also is a number between two previously suggested numbers, Euler (2.71) and Golden ratio (1.6), that underestimate and overestimate the number

of observed bands respectively. We changed the text to denote this limitation more clearly and explain the reasoning.

“It has long been noted that the bandwidth of neural rhythms is nearly doubled from one band to the next. Numbers between the Golden Ratio, 1.618 \citep{Roopun2008TemporalRhythms, Pletzer2010WhenEEG}, and the Euler number, 2.7182 \citep{Buzsaki2004NeuronalNetworks,Penttonen2003NaturalOscillators} have been suggested for this ratio between consecutive bands. The Golden Ratio, as the ratio between consecutive bands, seems to be an underestimation since it needs an introduction of sub-bands \citep{Pletzer2010WhenEEG} that are not widely reported and the Euler number seems to be an overestimation since it misses the alpha-band \citep{Buzsaki2004NeuronalNetworks}. So we decided to use two as a starting estimation point for the width of slow cycles that matches the experimental observations better and ensures the maximal efficacy of coupling across bands \citep{Klimesch2013AnCoupling}. However, it should be noted that further work needs to be performed to verify the validity and uniqueness of these patterns of activity (see discussion).”

In the Figure 5 legend, it is highly unusual to cite a link to figshare (Sabri 2023) that is specific to this paper. This is exactly why a supplemental section is included. If you don’t think it’s important enough for the supplemental section it might not be worth mentioning.

The reason for using an extra platform was that the number of panels for showing the spiking correlation patterns for all cycle types across all region pairs (for up to 20 regions in 3 animals) was more than can be included in the manuscript (~12k panels) and we think there might be helpful information in these panels for researchers focusing on different brain regions. However, we see the point that this is an unusual procedure and so we removed the citation from the main text and moved it to the legend of the supplementary figures that include the example correlation patterns as well as the ones that summarize the delays between brain areas across cycle types in case someone want to check the temporal dynamic of the correlation between specific brain regions for certain cycle types.

Either here or in the discussion, it’d be worth relating these delays to a biological mechanism. One possibility is they represent true delays in the propagation of neural signals between brain regions. Another is that one region inhibits the other, and there isn’t actually a delay in the sense of signal transfer speed.

Thank you so much for the insightful input. We changed the text to include these two possible biological mechanisms for the observed correlation patterns across regions.

“We observed specific temporal dynamics of correlated activity across brain regions, which depend on the time scales. For instance, as shown in \Cref{fig:fig5}, the two recording sites in TH and HPF are 600 ms delayed during the e cycles, but 3.8 s delayed during the $4e$ cycles. Such delays across regions might point to the propagation of the signal between regions or the inhibition of one region during the activation of the other region.”

Discussion

L248-258 is the motivation that I wish I had read in the introduction and really helps frame the subsequent analyses in the results. I highly encourage you to move this paragraph. The paragraph above provides important context, but I believe it can be condensed to 1-2 sentences when moved to the introduction (i.e. are oscillations "exhaust fumes").

We included the mentioned content in the updated text for the introduction, thank you for the suggestion:

"We hypothesize that the single cycles of rhythmic activity in neuronal spiking could be isolated if we examine the changes in the local population spike count: we pool the spikes from all neurons in a densely recorded population of neurons and measure the population spike rate across time as a univariate signal \citep{Okun2019DistinctTimescales, Okun2015DiverseCortex}. In densely recorded extracellular activity, where tens of neurons are recorded simultaneously within a local network, changes in the rate of population spike count reflect shared variations in the neuronal sub-threshold activity across the network. Sub-threshold activity has also been shown to follow LFP \citep{Haider2016MillisecondCortex, Perrenoud2016MembraneMice}. Taken together, these observations suggest that we can identify patterns of cyclic neural activity by analyzing the pooled spiking rate of the recorded population."

A very apparent future direction is applying a similar analysis at the single neuron level, and/or grouping spike rates of neurons according to some heuristic (location, cell-type/spike shape, tuning, etc.).

We added the following text to the discussion regarding this future direction:

"One future direction is to use spikes from functionally and/or anatomically distinct groups of neurons to identify the cyclic activity patterns. With this approach, we will be able to follow the propagation of the activity across the circuit."

In L271-L282, I definitely agree that detecting the optimal set of "neural basis functions" is a very important goal. As I've outlined above, I think it is important to show that existing tools are insufficient for this task (the AUC metric on the synthetic data would provide a clear result).

We added the comparison of cycle detection with spectral-based methods over synthetic signals as Fig S1-2.

In terms of identifying neural basis functions, it may be worth referencing that biophysical modeling is a promising approach towards this goal. The whole argument around characterizing aperiodic activity from Gao and Voytek builds from the idea that AMPA and GABA postsynaptic potentials have distinct time constants, and the shapes (basis functions) of these signals influence the 1/f slope even when there's no true rhythmicity. In a similar vein, biophysical models that attempt to understand the underlying mechanism of neural oscillations may prove to

be an indispensable tool for constructing these basis functions from "first principles", rather than generative models with no biological motivation. Below are some references of tools aimed at tackling this problem:

Lindén, H., Hagen, E., Łęski, S., Norheim, E. S., Pettersen, K. H., & Einevoll, G. T. (2014). LFPy: a tool for biophysical simulation of extracellular potentials generated by detailed model neurons. Frontiers in neuroinformatics, 7, 41.

Borges, F. S., Moreira, J. V., Takarabe, L. M., Lytton, W. W., & Dura-Bernal, S. (2022). Large-scale biophysically detailed model of somatosensory thalamocortical circuits in NetPyNE. Frontiers in Neuroinformatics, 16, 884245.
Neymotin, S. A., Daniels, D. S., Caldwell, B., McDougal, R. A., Carnevale, N. T., Jas, M., ... & Jones, S. R. (2020). Human Neocortical Neurosolver (HNN), a new software tool for interpreting the cellular and network origin of human MEG/EEG data. Elife, 9, e51214.

Dai, K., Gratiy, S. L., Billeh, Y. N., Xu, R., Cai, B., Cain, N., ... & Arkhipov, A. (2020). Brain Modeling ToolKit: An open source software suite for multiscale modeling of brain circuits. PLOS Computational Biology, 16(11), e1008386.

Similar to the above comment, for estimating neural basis functions in a purely data-driven approach, there is already some work on this in the EEG space which is very relevant:

Díaz, J., Ando, H., Han, G., Malyshevskaya, O., Hayashi, X., Letelier, J. C., ... & Vogt, K. E. (2023). Recovering Arrhythmic EEG Transients from Their Stochastic Interference. arXiv preprint arXiv:2303.07683.

Dupré la Tour, T., Moreau, T., Jas, M., & Gramfort, A. (2018). Multivariate convolutional sparse coding for electromagnetic brain signals. Advances in Neural Information Processing Systems, 31.

Thank you for pointing out these studies, we adopted the reviewer's phasing to include this line of research in the discussion and also cited the works on learning waveforms in the EEG:

"Next, we need to apply an unsupervised method to identify the optimal basis functions for reconstructing local population spiking and/or LFP \citep{Diaz2023RecoveringInterference,Dupre2018MultivariateSignals}. While data-driven approaches like these rely on generative modeling without direct biological motivation, another promising avenue of research focuses on biophysical modeling to uncover the mechanisms underlying neural oscillations and could become an essential tool for deriving these basis functions from fundamental principles \citep{Dai2020BrainCircuits,Borges2022Large-scaleNetPyNE,Linden2014LFPy:Neurons}."

Methods
Section 4.2

In L325, is “this monitor was derived at 180 Hz” correct? Or is this meant to say “this monitor was displayed at 180 Hz”

Corrected.

Section 4.3

One of the major contributions of this paper is a novel decoder-based analysis, but the details of this analysis are buried in a single paragraph in the methods. As highlighted above it was rather difficult to parse what was actually being used to train and evaluate the network. These issues can largely be mitigated by including typeset equations for 1) the synthetic data generation, and 2) the loss function used to train the LSTM. As it currently stands the text in the introduction/results/methods are all too vague to properly evaluate this method.

We changed the method section and added the suggested formulas for the synthesized signal used for the training and the loss function:

$$s_{\text{train}}(t) = \text{Normalize}\left(\sum_{i=1}^{7}\sum_{j=1}^{N_i} \alpha_{ij} c_{ij}(t - t_{ij})\right) + \eta(t)$$

where:

- $c_{ij}(t)$: The Gaussian function representing the j-th instance of the i-th cycle type, defined as:

$$c_{ij}(t) = \exp\left(-\frac{1}{2}\left(\frac{t}{\sigma_{ij}}\right)^2\right)$$

- σ_{ij}: The jittered standard deviation (width) for the j-th instance of the i-th cycle type, defined as:

$$\sigma_{ij} = \sigma_i + \Delta\sigma_{ij}, \quad \Delta\sigma_{ij} \sim \mathcal{N}(0, 0.3 \cdot \sigma_i)$$

  where $\sigma_i = \frac{d_i}{5}$.
- d_i: The base duration of the i-th cycle type, given by:

$$d_i = 2^{i-4} \cdot d_4$$

  Here, d_4 is the duration of the target cycle.
- d_{ij}: The jittered duration of the j-th instance of the i-th cycle type, related to σ_{ij} as:

$$d_{ij} = 5 \cdot \sigma_{ij}$$

- t_{ij}: The center time of the j-th instance of the i-th cycle type, ensuring that instances of similar cycle types don't overlap:

$$t_{ij} = t_{i(j-1)} + d_{i(j-1)}$$

  t_{4j} time points are the targets of decoding.
- α_{ij}: A binary inclusion variable sampled as:

$$\alpha_{ij} \sim \text{Bernoulli}(p), \quad p = 0.6$$

- Normalize$(\cdot)$: The normalization function scales the synthesized signal to lie between 0 and 1:

$$\text{Normalize}(s) = \frac{s - \min(s)}{\max(s) - \min(s)}$$

- $\eta(t)$: Additive Gaussian noise sampled as:

$$\eta(t) \sim \mathcal{N}(0, \sigma^2), \quad \sigma = 0.05$$

For training, the network output was set as a binary one-dimensional time series of the same length as the input. This output signal was set to zero everywhere except at the center of the target cycle.

$$y(t) = \begin{cases} 1, & \text{if } t = t_{4j} \text{ (center of the target cycle)} \\ 0, & \text{otherwise} \end{cases}$$

The sampling rate of the synthesized signal was set so that the target cycle was sampled 30 times per cycle. For predicting each output sample 61 samples from the input were provided to the network including 30 samples before and 30 samples after the target sample. A Mean Squared Error (MSE) loss function was used for the training:

$$\mathcal{L} = \frac{1}{T}\sum_{t=1}^{T}(y(t) - \hat{y}(t))^2$$

Minor Comments

In Figure 2B I would use red dots to denote the center of the cycle, orange is too similar to the heatmap colors

We changed the color of the dots to red.

In Figure 2D, why not denote the center of the second cycle?

The centers of the second cycles were also denoted with red dots.

Figure 2H seems to be a mistake and should be removed. Reordering should have no impact on the PSTH, and clearly this is identical to 2G.

We changed this panel to the distribution of the timing of the second sensory-driven gamma cycle.

Figure 2I-K, the caption description is mislabeled. I believe it should state "across trials with I. facial motion, J. gamma cycle amplitude… etc."

Corrected, thank you!

Figure 2M, asterisks should indicate if the decoding performance is significantly above chance

In comparison to the chance level, all the p-values are less than 0.0005 (for the center bin, p=6e-11), so we added this note to the caption.

Figure 3A-B: it would be useful to label something similar to "TH (mouse 1)" and "TH (mouse 2)" in the actual figure

We added the mouse number to the titles in panels A and B
