# Supplementary material for: Isolating single cycles of neural oscillations in population spiking
Source: PLoS Comput Biol. 2025 Jun 4;21(6):e1013084. doi: 10.1371/journal.pcbi.1013084 (PMC12136316; doi:10.1371/journal.pcbi.1013084)
Supplement: Attachment — Submitted filename: Response_to_Reviewers_auresp_2.pdf [file pcbi.1013084.s031.pdf]

Dear Editorial Committee,

We appreciate the opportunity to revise our manuscript based on the reviewers' thoughtful comments. We have carefully addressed concerns raised in this second round of review and made improvements to the manuscript that you can find below.

In response to Reviewer #1's concerns about the generalizability of our algorithm, we have:

- Added clarification about potential biases in the decoder's performance when applied to biological signals
- Emphasized the validation steps we took to ensure our findings reflect intrinsic characteristics of the neural data

In response to Reviewer #2's concerns, we have:

- Added quantitative metrics (AUC of ROC curves) to better demonstrate the improvements of our technique compared to existing methods
- Provided clearer explanations of how our cycle detection method outperforms peak detection approaches
- Made all suggested textual corrections and clarifications

We believe these revisions have strengthened the manuscript considerably and addressed the reviewers' concerns. We are grateful for their constructive feedback, which has helped us improve the clarity and rigor of our work.

Sincerely,

Ehsan Sabri and Renata Batista-Brito

**Reviewer #1:**
*Thanks for the point-by-point reply. I appreciate the efforts paid by the authors on revising the manuscript. Major concerns still remain. On validating the LSTM algorithm, the DL-driven models learn the distribution of input potentially in a manner not similar to how human understand the input. That is, no matter how biologically plausible the generated waves are, we cannot guarantee the learned parameters of LSTM can be generalizable on real data, unless validation can be performed on real data. The reviewer would suggest to show the performance of segmenting waves on real data. Otherwise, please modify the statement on the following parts by indicating that the findings enabled by the algorithm may be biased by the learned bias and the findings are not guaranteed to reflect the intrinsic characteristics within data. The findings may reflect the learning bias induced by generated data.*

We appreciate the reviewer’s feedback and the opportunity to clarify this point in the manuscript. While we matched the spectral distribution of the synthetic training signal to the neural data, other biological signal characteristics may still influence the decoder's performance. To address this, we conducted additional analyses and found that the detected cycles in population spiking independently reflect the known oscillatory activity in the LFP, as well as cross-frequency couplings widely reported in the literature. We have revised the introduction to highlight the potential bias of the decoder toward the training set.

“The decoder was trained on the synthetic signal that is matched to the spectral content of the biological signals. However, additional features not captured in the training signal could introduce bias when applying the decoder to real neuronal data. To assess the validity of the detected cycles in recorded data, we demonstrated that the average LFP around these cycles, identified in population spiking, exhibits similar cyclic patterns. Furthermore, we confirmed known neural cross-frequency couplings using these isolated cycles in population spiking.”

**Reviewer #2:**

***Summary***
*The authors have faithfully addressed my concerns from the first round of revisions. The paper reads much more clearly and highlights the strengths of this new method for characterizing an understudied representation of spiking activity. There are a few minor changes highlighted below that should be addressed before a final proof. Specifically the comparisons to existing techniques should be quantified with summary statistics to verify the claimed improvements. My recommendation is to accept with minor revisions.*

We thank the reviewer for the helpful comments, the manuscript reads much better after applying the suggested changes. Please find the details of modifications following the points below.

**Major Comments**
*For the comparisons made in Figure S1-2, AUC of ROC should be quantified to directly compare the techniques shown.*

*Figure S2-1 needs a quantitative comparison to demonstrate the improvement of your technique.*

We have added the difference of AUC of ROCs for the comparison with band-pass filtering in panel D of S1-2 and updated its caption as below. Since both methods produce bounded ROC curves that do not extend to False Alarm = 1, we calculated the partial Area Under the Curve (pAUC), normalized by the maximum False Alarm Rate of each method. This normalization ensures a fair comparison by measuring the average Hit Rate within each method's operational range.

“The partial Area Under the Curve (pAUC) of the ROC for both methods was calculated by normalizing to each method’s maximum False Alarm rate, as their ROC curves are bounded. isoCycle: 0.975 $\pm$ 0.002 and band pass filtering: 0.879 $\pm$ 0.005.”

*Further it is not clear visually how you are supposed to derive the conclusions in L186 when comparing to Figure 2.*

Thank you for the note. We have added the following explanation  to clarify this point:

“While using the peak timing of population spiking for reordering and jitter correction can partially capture gamma cycle dynamics, it does not accurately align trials. This is evident in

\ref{fig:s2_1}B, where the gamma band is less distinct compared to \ref{fig:fig2}B, and in \ref{fig:s2_1}G), where the jittered-corrected response does not return to baseline. Additionally, this method is less effective at distinguishing consecutive cycles. Specifically, the second gamma bands detected from the peak of population spiking span 60 ms - three times the typical gamma duration (\ref{fig:s2_1}H) - indicating that consecutive bands cannot be reliably separated by using the peak of population spiking. Cycle detection outperforms peak detection because consecutive cycles do not always manifest as distinct peaks."

**<u>Minor Comments</u>**

**Abstract**

- *L10: "...the densely recording of large neuronal…" -> "...the recording of large neuronal…". Or just change it to "the dense recording of large…".*

  Corrected.

- *L23: When you say you address "two key questions", the following aren't exactly questions. Perhaps "we achieved two main goals" or something to that nature. You could also rewrite these such that you define a specific question "precise alignment of sensory trials" addresses.*

  Thank you for raising this point, we rewrote this part to clarify:

  "1) How can we account for biological variation in neural signal transmission timing across trials during the sensory stimulation experiments? By isolating gamma cycles driven by sensory input, we achieved a more accurate trial alignment in the sensory stimulation experiments conducted in the primary visual cortex (V1) of mice. This alignment accounts for biological variability in sensory signal transmission times from the retina to V1 across trials, enabling a clearer understanding of neural dynamics in response to sensory stimuli. 2) How do spiking correlations across brain regions vary by timescale? We used the distinct spiking cycles in simultaneously recorded brain regions to examine the correlation of spiking across brain regions, separately for different timescales. Our findings revealed that the delays in population spiking between brain regions vary depending on the brain regions involved and the timescale of the oscillations."

**Introduction**

*L87: "...when pooled together spikes…" -> "...when pooling together spikes…"*

Corrected.

**Results**

- *For Figure 1G, it'd be worth adding a sentence describing what's shown in the CSD. I find it rather striking that the patterns of sinks and sources are completely different. For example gamma oscillations only show sinks in a specific layer, whereas for theta/delta cycles the sinks/sources alternate. This is clear evidence that these oscillations arise from distinct underlying mechanisms, and further strengthens the case for analyzing global spiking oscillations.*

Thank you for the suggestion. We have now added the following text regarding this point:

"We also compared the patterns of Current Source Density (CSD) corresponding to the averaged LFPs around the cycles detected from the population spiking (Fig \ref{fig:fig1}G). The distinct configurations of sources and sinks across different cycle types suggests that different mechanisms underly different cycle types."

- *L146: When referring to temporal delays in faster cycles (Figure 1F), are you suggesting that this is exclusive to faster rhythms, or is this common to both? This is not easily verified visually for slower rhythms because of the time axis, it could very well be the case that these delays are observed if you expand the axes.*

  We cannot rule out the possibility of millisecond-range temporal delays for slower cycles across layers, as our resolution is insufficient to detect such delays in slower cycles (the sampling rate of the algorithm is proportional to the cycle timescale, with the population firing rate sampled at 30 samples/target_cycle, varying for each cycle type). However, in slower (longer) cycles, millisecond-scale delays result in smaller phase differences between layers compared to faster cycles, making them less likely to have significant functional consequences.

- *L184: I believe you are referencing the wrong figure, Figure S2-1 is the one that performs this analysis*

  Corrected, thank you!

- *L185: "However, this method provides less accurate trial alignment and less precise differentiation between adjacent cycles compared to using the timing of individual cycles for jitter correction." This statement should be quantified, with a description of how it's quantified. As it stands I have a hard time confirming this statement visually by comparing it to Figure 2. Can you add some text explaining precisely how you derive this conclusion by inspecting the plots? It would be helpful to include the original data from Figure 2 to facilitate a side-by-side comparison.*

  We added the following explanation to make this point clear:

  "While using the peak timing of population spiking for reordering and jitter correction can partially capture gamma cycle dynamics, it does not accurately align trials. This is evident in \ref{fig:s2_1}B, where the gamma band is less distinct compared to \ref{fig:fig2}B, and in \ref{fig:s2_1}G), where the jittered-corrected response does not return to baseline. Additionally, this method is less effective at distinguishing consecutive cycles. Specifically, the second gamma bands detected from the peak of population spiking span 60 ms - three times the typical gamma duration (\ref{fig:s2_1}H) - indicating that consecutive bands cannot be reliably separated by using the peak of population spiking. Cycle detection outperforms peak detection because consecutive cycles do not always manifest as distinct peaks."

**Discussion**

- *L349: Regarding the assumptions in the second to last paragraph, it seems you would miss "cycles" that correspond to synchronous decreases in the firing of neurons when the data is generated by gaussians, a limitation worth addressing in the discussion as a significant amount of LFP activity is attributed to inhibitory synaptic activity.*

  Thank you for this point, we added it at the end of this paragraph:

  "Another assumption that requires further investigation is the absence of inverse cyclic activity in our model, which would represent the net inhibitory input to the network that reduces the probability of population spiking with a cyclic pattern."

**Methods**
- *5.3 Cycle Detection: "a Gausinna noise layer" -> "a Gaussian noise layer"*

  Corrected.

**Figures**
- *Figure S1-2: Area under curve of ROC should be reported in figure legend*

  Added.

  "The partial Area Under the Curve (pAUC) of the ROC for both methods was calculated by normalizing to each method's maximum False Alarm rate, as their ROC curves are bounded. isoCycle: 0.975 $\pm$ 0.002 and band pass filtering: 0.879 $\pm$ 0.005."
  .

- *Figure 2M: "ttest" -> "t-test"*

  Corrected, thank you!
