# Supplementary material for: Isolating single cycles of neural oscillations in population spiking
Source: PLoS Comput Biol. 2025 Jun 4;21(6):e1013084. doi: 10.1371/journal.pcbi.1013084 (PMC12136316; doi:10.1371/journal.pcbi.1013084)
Supplement: Attachment — Submitted filename: ploscompbio_revision_comments.docx [file pcbi.1013084.s029.docx]

**Summary**

The authors have faithfully addressed my concerns from the first round of revisions. The paper reads much more clearly and highlights the strengths of this new method for characterizing an understudied representation of spiking activity. There are a few minor changes highlighted below that should be addressed before a final proof. Specifically the comparisons to existing techniques should be quantified with summary statistics to verify the claimed improvements. My recommendation is to accept with minor revisions.

**Major Comments**

For the comparisons made in Figure S1-2, AUC of ROC should be quantified to directly compare the techniques shown.

Figure S2-1 needs a quantitative comparison to demonstrate the improvement of your technique. Further it is not clear visually how you are supposed to derive the conclusions in L186 when comparing to Figure 2.

**Minor Comments**

**Abstract**

- L10: “...the densely recording of large neuronal…” -> “...the recording of large neuronal…”. Or just change it to “the dense recording of large…”.
- L23: When you say you address “two key questions”, the following aren’t exactly questions. Perhaps “we achieved two main goals” or something to that nature. You could also rewrite these such that you define a specific question “precise alignment of sensory trials” addresses.

**Introduction**

L87: “...when pooled together spikes…” -> “...when pooling together spikes…”

**Results**

- For Figure 1G, it’d be worth adding a sentence describing what’s shown in the CSD. I find it rather striking that the patterns of sinks and sources are completely different. For example gamma oscillations only show sinks in a specific layer, whereas for theta/delta cycles the sinks/sources alternate. This is clear evidence that these oscillations arise from distinct underlying mechanisms, and further strengthens the case for analyzing global spiking oscillations.
- L146: When referring to temporal delays in faster cycles (Figure 1F), are you suggesting that this is exclusive to faster rhythms, or is this common to both? This is not easily verified visually for slower rhythms because of the time axis, it could very well be the case that these delays are observed if you expand the axes.
- L184: I believe you are referencing the wrong figure, Figure S2-1 is the one that performs this analysis
- L185: “However, this method provides less accurate trial alignment and less precise differentiation between adjacent cycles compared to using the timing of individual cycles for jitter correction.” This statement should be quantified, with a description of how it’s quantified. As it stands I have a hard time confirming this statement visually by comparing it to Figure 2. Can you add some text explaining precisely how you derive this conclusion by inspecting the plots? It would be helpful to include the original data from Figure 2 to facilitate a side-by-side comparison.

**Discussion**

- L349: Regarding the assumptions in the second to last paragraph, it seems you would miss “cycles” that correspond to synchronous decreases in the firing of neurons when the data is generated by gaussians, a limitation worth addressing in the discussion as a significant amount of LFP activity is attributed to inhibitory synaptic activity.

**Methods**

- 5.3 Cycle Detection: “a Gausinna noise layer” -> “a Gaussian noise layer”

**Figures**

- Figure S1-2: Area under curve of ROC should be reported in figure legend
- Figure 2M: “ttest” -> “t-test”
